# Negative Thermal Expansion Near the Precipice of Structural Stability in Open Perovskites

**DOI:** 10.3389/fchem.2018.00545

**Published:** 2018-11-20

**Authors:** Connor A. Occhialini, Gian G. Guzmán-Verri, Sahan U. Handunkanda, Jason N. Hancock

**Affiliations:** ^1^Department of Physics, University of Connecticut, Storrs, CT, United States; ^2^Institute of Materials Science, University of Connecticut, Storrs, CT, United States; ^3^Centro de Investigación en Ciencia e Ingeniería de Materiales, Universidad de Costa Rica, San José, Costa Rica; ^4^Materials Science Division, Argonne National Laboratory, Argonne, IL, United States

**Keywords:** negative thermal expansion, structural negative thermal expansion, quantum phase transition, structural phase transition, perovskite, antiferrodistortive phase transition, scandium trifluoride

## Abstract

Negative thermal expansion (NTE) describes the anomalous propensity of materials to shrink when heated. Since its discovery, the NTE effect has been found in a wide variety of materials with an array of magnetic, electronic and structural properties. In some cases, the NTE originates from phase competition arising from the electronic or magnetic degrees of freedom but we here focus on a particular class of NTE which originates from intrinsic dynamical origins related to the lattice degrees of freedom, a property we term *structural* negative thermal expansion (SNTE). Here we review some select cases of NTE which strictly arise from anharmonic phonon dynamics, with a focus on open perovskite lattices. We find that NTE is often present close in proximity to competing structural phases, with structural phase transition lines terminating near *T*=0 K yielding the most prominent displays of the SNTE effect. We further provide a theoretical model to make precise the proposed relationship among the signature behavior of SNTE, the proximity of these systems to structural quantum phase transitions and the effects of phase fluctuations near these unique regions of the structural phase diagram. The effects of compositional disorder on NTE and structural phase stability in perovskites are discussed.

## 1. Introduction

**Figure 1:**
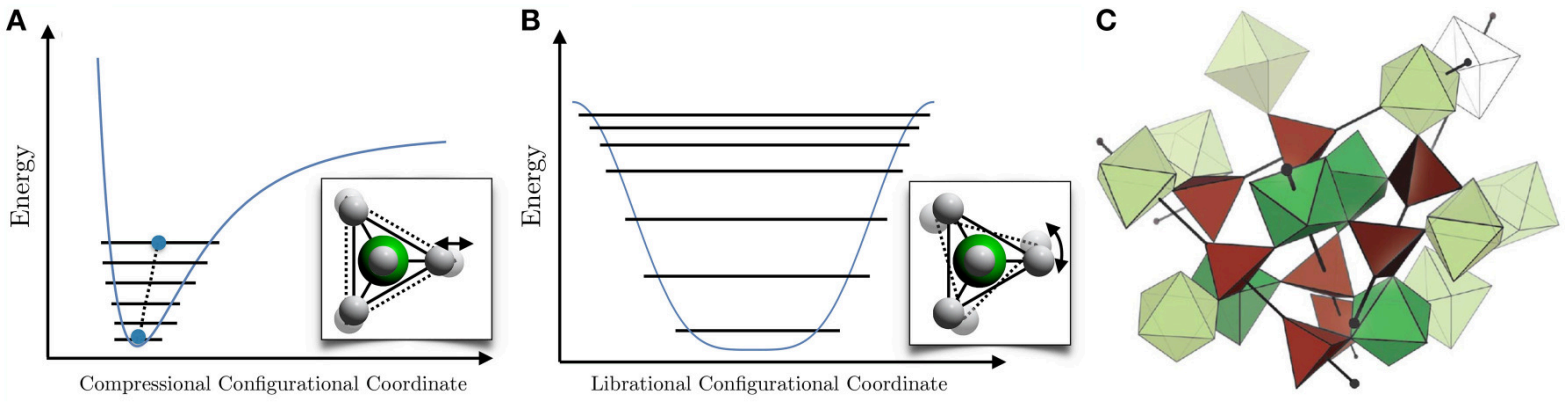
Intermolecular potentials provide a heuristic explanation for the common occurrence of **(A)** PTE and **(B)** NTE. **(C)** The complex structure of the low-symmetry α-phase of ZrW_2_O_8_, from Hancock et al. (2004a).

Thermal expansion is among the most widely recognized thermodynamic properties of materials. From a textbook perspective (Ashcroft and Mermin, 1976), thermal expansion occurs through anharmonic free energy terms arising from nuclear lattice degrees of freedom. The dominant appearance of the positive thermal expansion (PTE) found in both research-grade and industrial materials is heuristically ascribed (Barrera et al., 2005; Miller et al., 2009; Takenaka et al., 2012) to the expected anharmonic behavior of a generic interatomic potential, which is hard at short distance and soft at large distance (Figure 1A). As temperature is raised, higher energy excitations are populated which have an ever increasing mean separation, dilating the bond and presumably lattice dimensions. Of course this is not a theorem any more than crystals are molecules and collective motion of lattices permit various potential landscapes, such as a librational coordinate of tetrahedral molecular solids (Prager and Heidemann, 1997), which possess clear qualitative differences (Figure 1B).

Mention of negative thermal expansion (NTE), a material's tendency to shrink when heated, often evokes discussion of liquid water-ice expansion responsible for icebergs and the 4K temperature window above the ice-water phase boundary where phase fluctuations occur. This is an example of a route to achieving NTE which relies on broadened phase transitions between a low-temperature high-volume phase fluctuating into a high-temperature low-volume phase, other examples of which include the industrial alloy InVar (Guillaume, 1920) (Fe_64_Ni_36_) and more recently discovered NTE materials (Takenaka and Takagi, 2005; Azuma et al., 2011; Qu et al., 2012; Chen et al., 2013a,b) (for more details on this approach, see Takenaka's review in this volume Takenaka, 2018). While this route to realizing NTE is promising for many applications requiring only dimensional concerns, NTE at these broadened transitions occurs only in heavily restricted regions of the magnetic and electronic phase diagrams, constraining a thermodynamic number of degrees of freedom to achieve a single mechanical characteristic. Thus, these types of NTE materials will be severely restricted in their potential for multifunctional applications.

Remarkably, there exists a growing class of materials with strong, isotropic, robust, and thermally persistent NTE that arises from structural motifs (Martinek and Hummel, 1968; Mary et al., 1996; Evans et al., 1997a,b; Pryde et al., 1997; Ernst et al., 1998; Perottoni and Jornada, 1998; Ramirez and Kowach, 1998; Ramirez et al., 2000; Mittal et al., 2001, 2004, 2018; Cao et al., 2002, 2003; Ouyang et al., 2002; Drymiotis et al., 2004; Hancock et al., 2004b; Kennedy and White, 2005; Lee et al., 2005; Tucker et al., 2005, 2007; Pantea et al., 2006; Figueirêdo and Perottoni, 2007; Han and Goddard, 2007; Keen et al., 2007, 2011; Schlesinger et al., 2008; Zhou et al., 2008; Greve et al., 2010; Gallington et al., 2013, 2014; Gupta et al., 2013; Morelock et al., 2013b; Bridges et al., 2014; Sanson, 2014). NTE in these systems is often discussed in connection with transverse fluctuations of a linkage between volume-defining vertices, which may accompany the librational, or hindered rotational motion of polyhedral subunits. The energy landscape for such motion tends to be much softer (0–2 THz) than bond-stretching motion (10–30 THz in oxides) which is often the implicated culprit of PTE. Here, NTE arises from the cooperative fluctuations of the bond network on THz time scales under very strong anharmonic influences and appears without necessarily constraining the magnetic or electronic phase diagram, permitting one to envisage new multifunctional materials with diverse mechanical, spin, orbital, thermal, electronic, superconducting, and more exotic order coexisting with NTE. Study of the unusual physics behind this type of NTE informs discovery efforts to find new contexts for this remarkable phenomenon. In addition, NTE materials hold promising application potential in stabilizing fiber Bragg gratings for high-speed telecommunication (Fleming et al., 1997; Kowach and Ramirez, 2002), substrates for devices which benefit from thermally controlled stresses and the formation of rigid composite structural materials with engineered thermal characteristics through combinations of PTE and NTE components (Balch and Dunand, 2004; De Buysser et al., 2004; Lommens et al., 2005; Sullivan and Lukehart, 2005; Lind et al., 2011).

This second circumstance for NTE, which we term *structural* NTE (SNTE), is the focus of the present article. The field of SNTE has been met with sustained interest from the physics, chemistry, and materials science communities since the re-discovery of the strong SNTE in ZrW_2_O_8_ in 1996 (Martinek and Hummel, 1968; Mary et al., 1996). The SNTE effect here persists over the temperature range 4-1050 K and has a sizable linear coefficient of thermal expansion (CTE) of α_ℓ_ ≃ −9 ppm/K near room temperature, which is isotropic due to the cubic symmetry maintained at all observed temperatures under ambient pressure. The low-symmetry α-phase structure of ZrW_2_O_8_ (Figure 1C) consists of ZrO_6_ octahedra and WO_4_ tetrahedra in the *P*2_1_3 space group, which has a screw axis along [111]. An order-disorder structural transition to a (cubic) $Pm\bar{3}$ γ-phase occurs at zero pressure and *T*_*c*_ ≃ 450K. The NTE effect survives the structural transition, with a small discontinuity and reduction in the CTE to α_ℓ_ ≃ −6 ppm/K. Furthermore, application of hydrostatic pressure at *T* = 300K first induces an orthorhombic transition at *P*_*c*_ = 0.3 GPa, followed by pressure-induced amorphization realized between *P* = 1.5−3.5 GPa (Evans et al., 1997b; Perottoni and Jornada, 1998; Ravindran et al., 2001). Both the α- and γ-phases contain four formula units, *N* = 44 atoms, in each unit cell, leading to a complex phononic structure with 3 acoustic and 3*N* − 3 = 129 optical branches.

Despite decades of intense research, the complex structure and associated dynamics of the ZrW_2_O_8_ lattice and the related *MA*_2_O_8_ compounds complicates the interpretation of both theoretical and experimental investigations into the mechanisms of SNTE. For instance, a commonly identified feature in the low-temperature α-phase is the two WO_4_ tetrahedra with unshared “terminal” oxygen atoms aligned along the screw axis. The under-constrained freedom of these tetrahedra along this axis is often cited as being responsible for the softness of the crucial NTE modes, but there is much debate as to the precise nature of the mode and its contributions to NTE (Ramirez et al., 2000; Hancock et al., 2004b). Several attempts at describing the soft mode as either a translation or rotation of the WO_4_ tetrahedron were addressed via the space group symmetry—both rotational and translational motion are permitted and necessarily coupled due to the lost inversion symmetry. Another level of controversy in ZrW_2_O_8_ is the extent to which the molecular subunits may be regarded as rigid (Cao et al., 2002; Tucker et al., 2007; Bridges et al., 2014; Sanson, 2014; Dove and Fang, 2016). Although ZrW_2_O_8_ presents clear scientific challenges, its discovery is significant in that it ignited a flurry of research into the microscopic origins of the SNTE, both theoretical and experimental, employing both thermodynamic (Ramirez et al., 2000) and spectroscopic (Ernst et al., 1998; Drymiotis et al., 2004; Hancock et al., 2004b; Pantea et al., 2006) probes of the low-energy lattice behavior. Some essential, guiding observations were revealed during the ensuing years: (i) ZrW_2_O_8_ has unusually low-energy lattice modes near 2-3meV (Ramirez et al., 2000; Hancock et al., 2004b), (ii) structural phase transitions are readily induced via light hydrostatic pressure (Evans et al., 1997b; Perottoni and Jornada, 1998; Ravindran et al., 2001) and (iii) the SNTE arises from a delicate balance of the degrees of freedom and constraint in the host lattice framework (Cao et al., 2002; Tucker et al., 2007; Bridges et al., 2014; Sanson, 2014; Dove and Fang, 2016).

One central question motivating SNTE research is why some materials show SNTE and others do not? To address this question will open avenues to discovery of new NTE materials and advancing technology born from its unique properties. While the precise mechanisms behind the dramatic SNTE in ZrW_2_O_8_ are still under contention, a variety of other simpler systems with equally impressive SNTE have been discovered in recent years (Rodriguez et al., 2009; Greve et al., 2010; Hancock et al., 2015). In moving toward the goal of a deeper understanding of SNTE mechanisms, we sharpen our focus on the growing class of perovskite materials exhibiting NTE, including ScF_3_, ReO_3_ and related structural family members. We consider the rich structural phase diagrams of the perovskite structure and their description in terms of octahedral tilts and the corresponding slow lattice dynamics associated with the structural transitions. Although numerous, the hierarchy of phases is well understood and documented, making perovskites a particularly simple framework on which to study the interplay of lattice dynamics and macroscopic phenomena like NTE. In particular, we note how the corresponding dynamic modes of the perovskite lattice relate to soft-mode instabilities that accompany the approach to realized and incipient structural phase transitions and how these are coupled to mechanisms resulting in SNTE. Most importantly, we further develop the apparent connection between the emergence of SNTE alongside phase fluctuations that occur near *T*=0K structural quantum phase transitions (SQPTs), for which we present the available experimental evidence and develop a systematic modeling scheme to explain the coupling between phase fluctuations and thermal expansion anomalies in perovskite materials.

## 2. Perovskites, structural phases and soft-mode induced transitions

**Figure 2:**
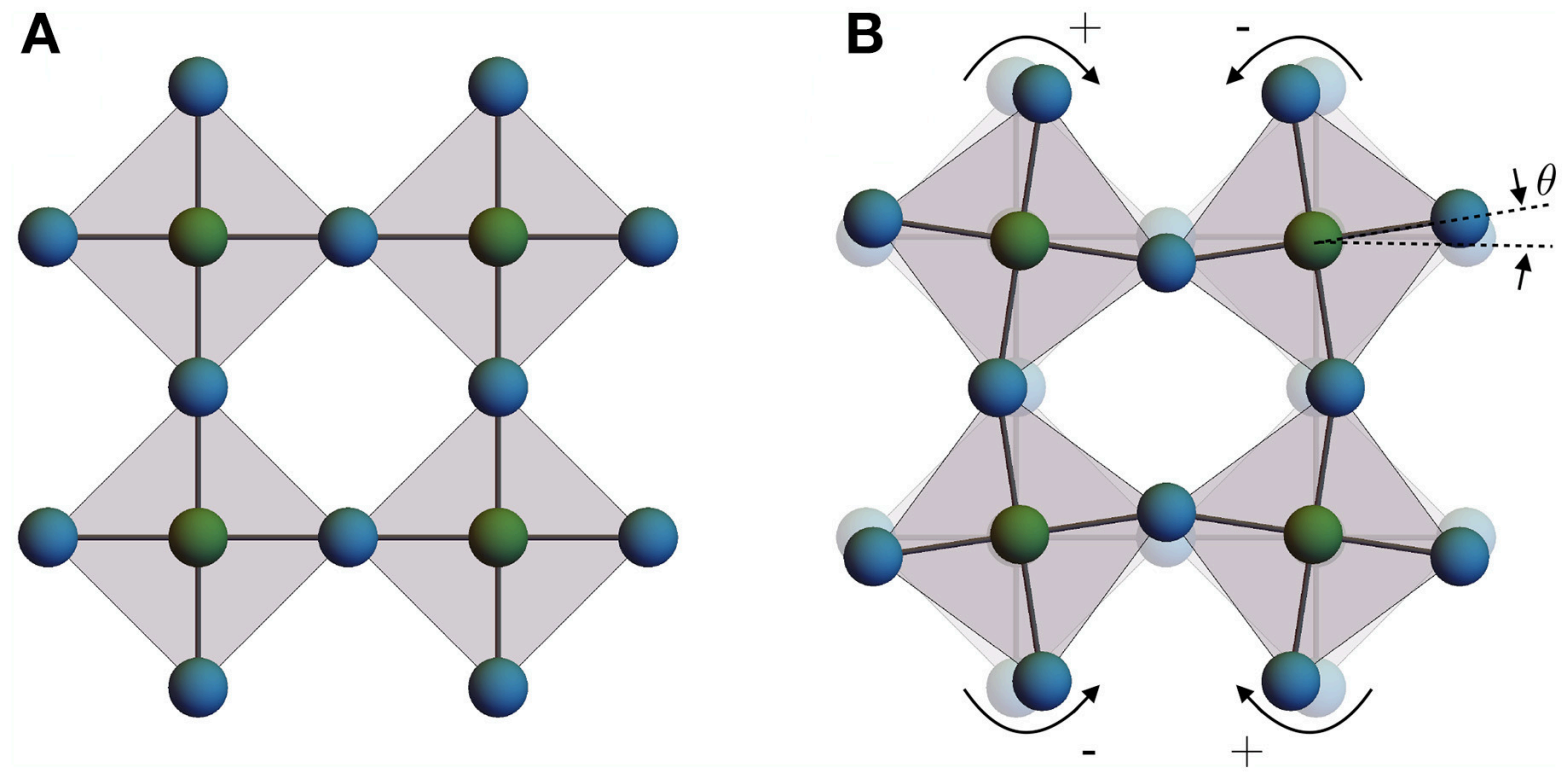
**(A)** A single 2D layer of perovskite octahedra in the high-symmetry cubic phase, viewed along one of the 3 fourfold axes and **(B)** the same layer with a non-zero tilt angle θ, showing the constrained motion of neighboring octahedra due to shared inter-octahedral *X*-sites.

**Figure 3:**
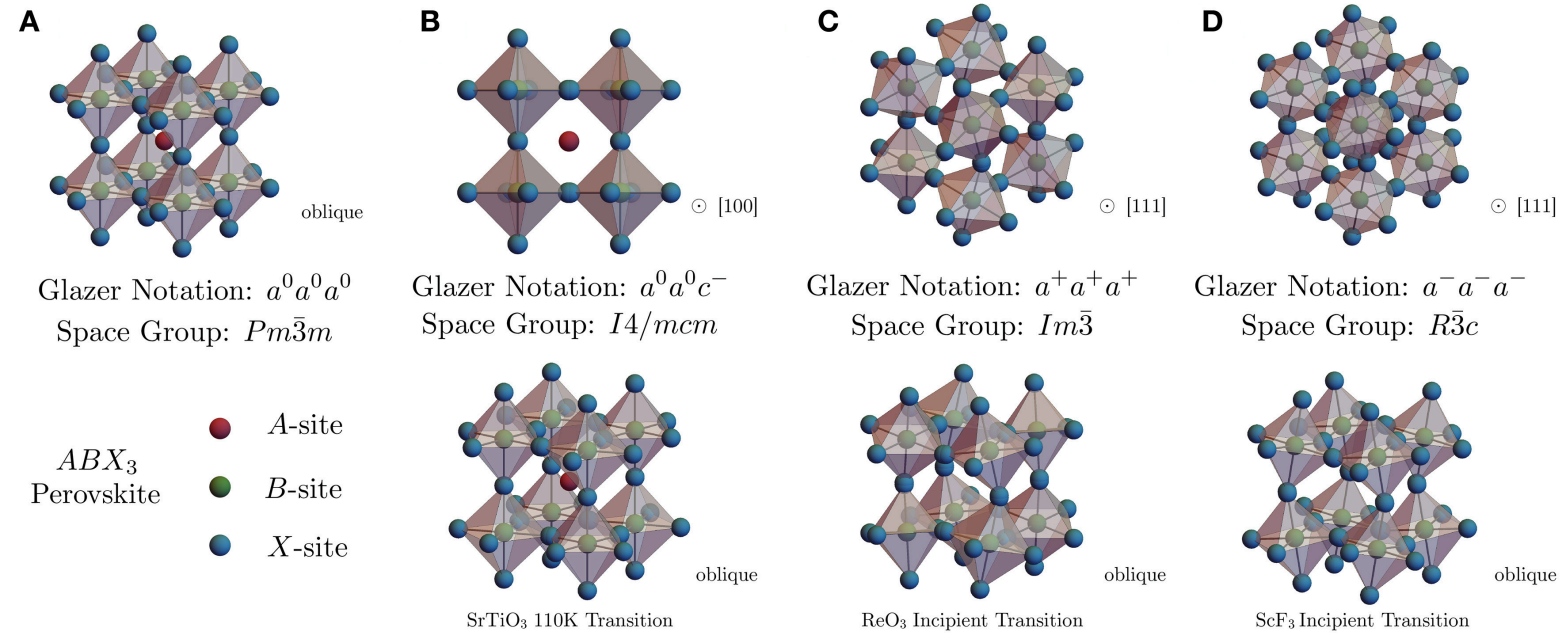
Shown here is **(A)** the general perovskite structure of formula unit *ABX*_3_ (see text), in the highest symmetry cubic space group $Pm\bar{3}m$ corresponding to a Glazer tilt notation *a*^0^*a*^0^*a*^0^. Also shown are common octahedral tilt lower-symmetry perovskites found in **(B)** the SrTiO_3_ *T*_*c*_ = 110K $Pm\bar{3}m$ to *I*4/*mcm* tetragonal structural transition **(C)** the triply degenerate $M_{3}^{+}$ phonon condensation in the low-*T*, high-*P* ReO_3_ structural transition (see text) and **(D)** the triply degenerate $R_{4}^{+}$ phonon condensation responsible for the rhombohedral transition is *B*F_3_ open perovskite 3*d*-transition metal trifluorides which also acts as the dynamic soft-mode rotations in ScF_3_, corresponding to Glazer tilts of *a*^0^*a*^0^*c*^−^, *a*^+^*a*^+^*a*^+^, and *a*^−^*a*^−^*a*^−^, respectively.

The perovskite lattice structure may well be identified as the double-helix of the solid state–a framework which is highly functionalizable, tunable, robust, and underpins perhaps every known category of physical behavior. This includes high-temperature superconducting, itinerant ferromagnetic, local ferromagnetic, ferroelectric, insulating, metallic, glassy, as well as a plethora of antiferromagnetic and other poorly understood phases which appear to compete, coexist, and cooperate within typically rich and complex phase diagrams (Kimura et al., 2002; Maekawa et al., 2004; Takagi et al., 2010; Ngai et al., 2014). The cubic perovskites are lattice structures with formula unit *ABX*_3_, where the *A*-site is typically an alkali or alkaline earth metal ion, *B* is a transition metal and *X* is the anion, most commonly forming an oxide or a halide. The highest-symmetry solid phase is shown below in Figures 2A, 3A, with a cubic space group symmetry $Pm\bar{3}m$ and the *B*-site ions in an *n* = 6 octahedral coordination environment of *X*-site anions. A hierarchy of structural phases in the perovskites are achieved through various concerted rotations of the *BX*_6_ coordination octahedra. These phases have been cataloged and a relationship between octahedral tilts and the lower-symmetry space groups due to these structural distortions have been developed (Glazer, 1972; Glazer and IUCr, 1975) and are well-known in the ferroelectric community (Benedek and Fennie, 2013).

The scheme for indexing the possible octahedral tilts begins with a 2 × 2 × 2 unit cell of the cubic $Pm\bar{3}m$ perovskite and considers rotations of the octahedra about each of the 3 fourfold (*C*_4_) axes of the cubic phase. In the plane normal to a given rotation axis, neighboring octahedra are constrained to rotate at equal angles (θ) of opposite sign, since neighboring *B*-sites are bonded to a common *X*-site anion (Figure 2B); there is, however, a choice in the phase of rotations for columns of octahedra along the rotation axis. Which phase pattern is realized is denoted by a superscript of + or − for in- and out-of-phase stacking, respectively, or a superscript of 0 indicating a null rotation. The equality of rotation angles around each axis is given by using repeated characters. For instance, in Glazer notation *a*^+^*b*^+^*c*^+^ represents three unequal rotations about [100], [010] and [001], with all rotations in phase along each respective axis. Overall, there are 23 distinct possibilities of perovskite space groups and octahedral tilting patterns, which can be cubic to triclinic and anything in between. Several relevant examples of perovskite distortions and the Glazer notation are given in Figure 3.

**Figure 4:**
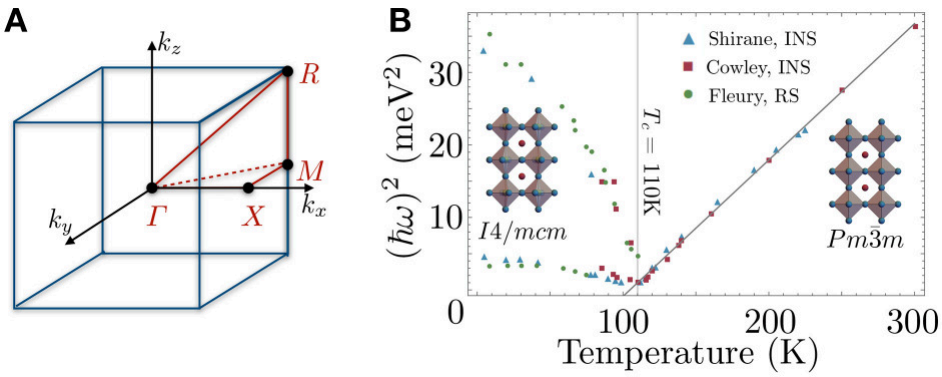
**(A)** The cubic Brillouin zone indicating the high-symmetry reciprocal lattice points Γ, *X*, *M* and *R*. **(B)** *R*-point mode softening on the approach to the *T*_*c*_ ≃ 110K structural transition in SrTiO_3_, measured from neutron (Cowley et al., 1969; Shirane and Yamada, 1969) and Raman (Fleury et al., 1968) scattering. Black line for *T*≥*T*_*c*_ shows agreement with the predicted soft-mode frequency for a dynamically driven second-order phase transition as given in Equation (1).

One of the best-studied structural instabilities in a perovskite structure is the transition at *T*_*c*_ ≃ 110K in SrTiO_3_, first identified with electron spin resonance (ESR) spectra by Unoki and Sakudo (Unoki and Sakudo, 1967) and later confirmed by many others (Cowley, 1964; Fleury et al., 1968; Cowley et al., 1969; Shirane and Yamada, 1969) via inelastic neutron scattering (INS), X-ray diffraction and Raman spectroscopy (RS). The room-temperature structure of SrTiO_3_ is that of the common $Pm\bar{3}m$ space group depicted in Figure 3A, but signatures of tetragonal symmetry in the ESR and Raman (Fleury et al., 1968) spectra are observed below *T* ≃ 110K, along with anomalies in the elasticity (Bell and Rupprecht, 1963). Details of the atomic displacements reveal the lower-symmetry structure is the tetragonal *I*4/*mcm* space group, which corresponds to a [001]-phase-staggered rotation of the TiO_6_ octahedra about a [001] rotation axis, that is an octahedral tilting pattern of *a*^0^*a*^0^*c*^−^ (Figure 3B). The displacements are related to the polarization of a zone-boundary optical phonon (irrep. *R*_25_) existing at the *R*-point of cubic Brillouin zone (BZ) (Figure 4A). In real space, the lowered-symmetry results in an effective doubling of the unit cell dimensions along one axis. In reciprocal space, however, the symmetry lowering occurs through a halving of the Brillouin zone and results in formation of new Bragg peaks as seen in an elastic scattering pattern (X-ray, neutron, electron). Dynamically, one can associate the transition to a slowing down of an optical phonon near the *R* (π*ππ*) point at the corner of the cubic Brillouin zone, corresponding to a “freezing” or “condensation” of one component of the triply degenerate *R*-point “soft” mode.

SrTiO_3_ is the first material in which soft modes were measured using inelastic scattering, and their concomitance with structural phase transitions was subsequently established through their observation in many other perovskites, e.g., LaAlO_3_, KMnF_3_, PbTiO_3_, and BaTiO_3_ (Shirane, 1974). A *soft-mode* can generally be defined as any normal mode of the dynamic lattice whose energy or, equivalently, frequency of vibration decreases anomalously. When such a vibrational frequency reaches ℏω = 0, the lattice becomes structurally unstable with respect to the displacements of this normal mode, and a subsequent symmetry-lowering, static deformation occurs to restore stability. For the simplest case of Landau-Ginsburg-Devonshire theory treated at the mean-field level, one expects a temperature dependence for the soft mode frequency (Scott, 1974; Shirane, 1974; Cowley, 1980):

(1) $$\begin{array}{r} \omega_{s}\left(T\right)\propto \sqrt{\left|T-T_{c}\right|} \end{array}$$

This dependence for the *R*-point soft-mode in SrTiO_3_ is shown in Figure 4B. This transition can be described by an order parameter, a quantity that is zero above and develops non-zero average values below *T*_*c*_, which follows the angle of rotation of the TiO_6_ octahedra about the principal axis in the low-symmetry tetragonal structure. The transition in SrTiO_3_ is, by all experimental accounts, second-order (continuous) in nature, but for many structural phase transitions signatures of the more common first-order (discontinuous) behavior renders the soft-mode approach invalid *a priori*. Nonetheless, soft modes can be used to interpret weakly first-order transitions and their frequency can be indicative of an incipient transition due to soft-mode coupling to other, primary order parameters. The 110K transition in SrTiO_3_ is also a prototypical example of critical behavior that can emerge in the vicinity of a structural transition, most notably the “central-peak” phenomenon discovered through an anomalous quasi-elastic peak in INS energy-transfer spectra, which can be explored elsewhere (Halperin and Varma, 1976; Topler et al., 1977; Riste et al., 1993).

In extreme cases, a material can approach dynamic instability with lowering temperature to near-zero soft mode energy, yet no temperature-induced transition is observed. In this situation, subsequent application of pressure, introduction of compositional disorder (doping) or other non-thermal parameters can perturb the ground-state of the system to drive the transition at *T* = 0K, realizing a quantum phase transition (QPT) (Sachdev and Keimer, 2011). Research surrounding the breakdown of canonical physical behavior near these quantum critical points (QCPs) is interesting in its own right (Coleman and Schofield, 2005; Gegenwart et al., 2008) but we below focus on QCPs within the structural phase diagrams and their relationship to the development of SNTE in a subset of the perovskites.

## 3. NTE in perovskite frameworks

**Figure 5:**
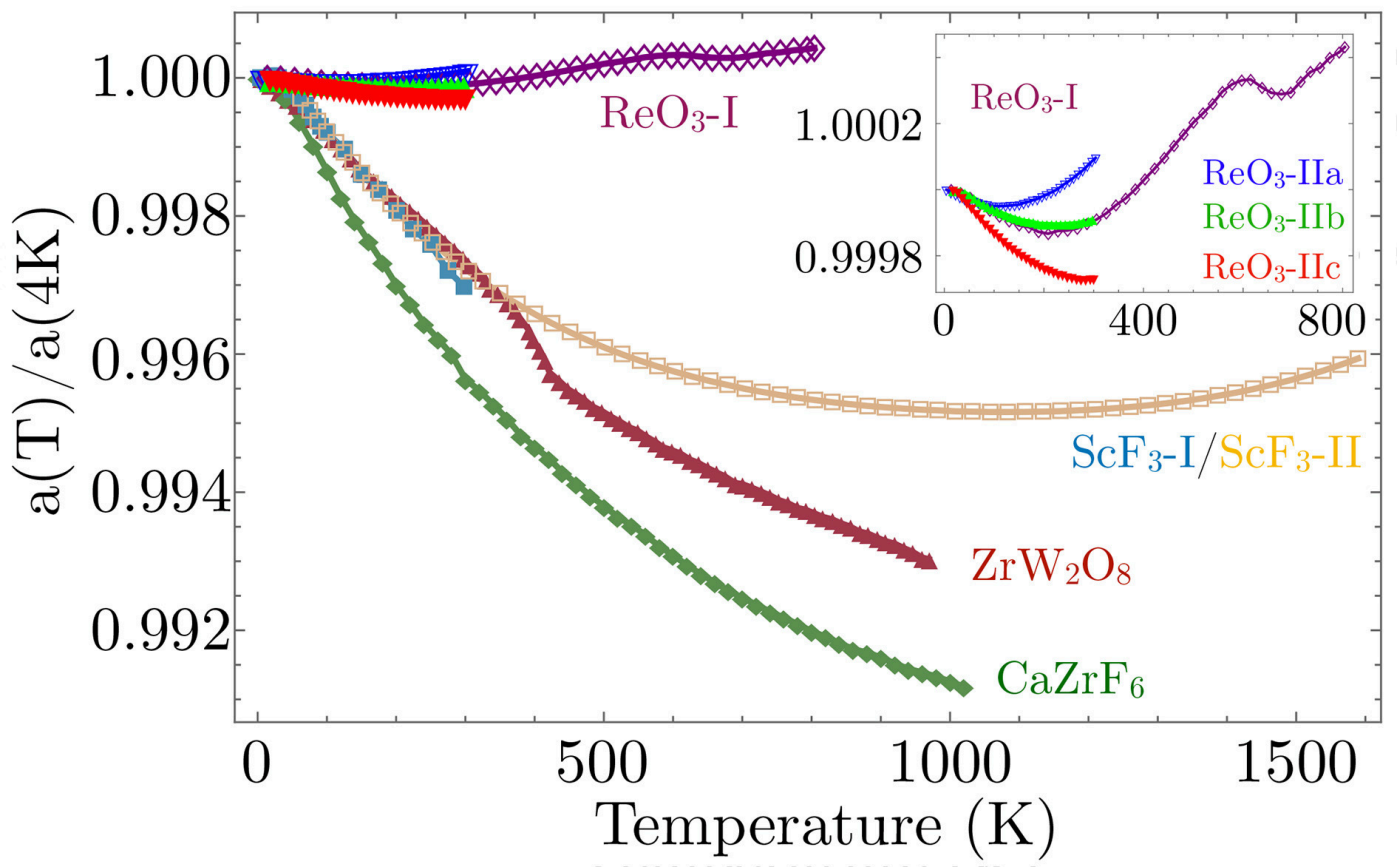
Shown here is the normalized cubic lattice parameter temperature dependence *a*(*T*)/*a*(4*K*) for ReO_3_, ScF_3_, and CaZrF_6_ NTE perovskites with strong SNTE material ZrW_2_O_8_ for comparison. Inset focuses on available data for ReO_3_ on 4 different samples, one (ReO_3_-I) over an extended temperature range (Chatterji et al., 2009b) and the other 3 (ReO_3_-II a-c) over a smaller temperature range, investigating the effects of compositional disorder (Rodriguez et al., 2009) (see section 5). Data taken from: ZrW_2_O_8_ (Mary et al., 1996), CaZrF_6_ (Hancock et al., 2015), ScF_3_-I (Handunkanda et al., 2015), ScF_3_-II (Greve et al., 2010), ReO_3_-I (Chatterji et al., 2009b), and ReO_3_-II (Rodriguez et al., 2009).

Most oxide perovskites *AB*O_3_ form with an *A*-site, otherwise requiring a rare hexavalent electronic configuration for charge balance. One prominent exception is ReO_3_, which forms with no *A*-site and maintains its cubic $Pm\bar{3}m$ space group symmetry down to the lowest measured temperatures. In addition, ReO_3_ has been known to exhibit SNTE for many years, which is often attributed to soft modes permitted by the open-perovskite (*A*-site-free) structure. The lack of the *A*-site puts fewer dynamical constraints on the motion of the ReO_6_ octahedra in comparison to the constraints imposed by the *A*-site in other perovskites. This permits large anisotropic thermal displacements of the linking oxygen atoms perpendicular to the Re-O-Re bond direction, making ReO_3_ more susceptible to lattice instabilities corresponding to these octahedral tilt patterns. This openness to the structure has also been noted as a key feature in many other SNTE materials, including ZrW_2_O_8_, leading to a larger set of soft, low-energy phonons that have mainly been identified as the cause of SNTE. Reports on the size of the SNTE effect in ReO_3_ vary, but in one report, SNTE was observed in two separated temperature windows of 2–220 K and 600–680 K (Chatterji et al., 2009b) with a maximum measured linear thermal CTE of α_ℓ_ = −2.56 ppm/K (Dapiaggi and Fitch, 2009) (Figure 5).

**Figure 6:**
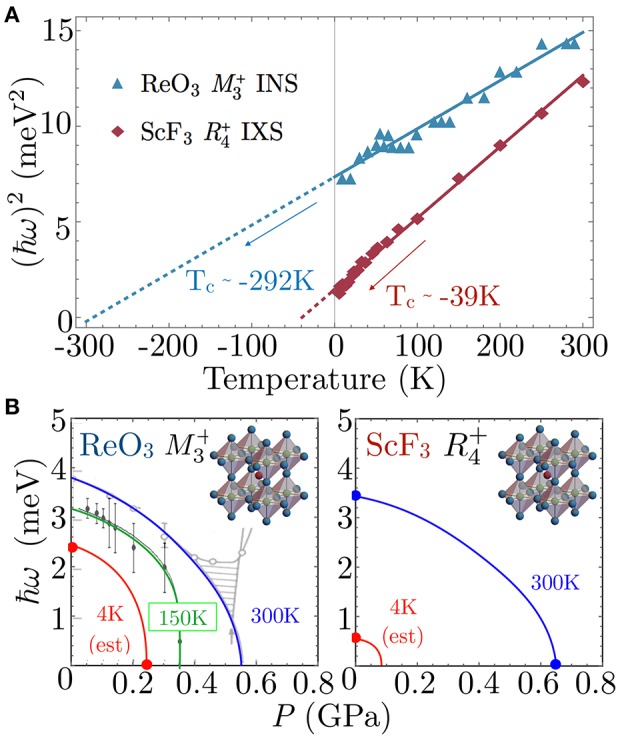
**(A)** Shows the squared energy (ℏω)^2^ of the $M_{3}^{+}$ and $R_{4}^{+}$ soft modes vs. temperature at ambient pressure in ReO_3_ and ScF_3_, respectively. As indicated, extrapolation by mean-field approximation (Equation 1) indicates structural transition temperatures at ambient pressure of *T*_*c*_ ≃ −292K for ReO_3_ and *T*_*c*_ ≃ −39K for ScF_3_. **(B)** Shows the soft mode energy vs. pressure at various temperatures for (left) the $M_{3}^{+}$ mode in ReO_3_ and (right) the $R_{4}^{+}$ mode in ScF_3_. Solid lines are guide to the eye and symbols in both **(A,B)** are taken from references (Axe et al., 1985; Chatterji et al., 2009a; Handunkanda et al., 2015). Comparison supports our assignment that ScF_3_ is closer to a SQPT than ReO_3_.

ReO_3_ undergoes several structural phase transitions under hydrostatic pressure and is most studied at room temperature. Early INS investigations at ambient temperature established that ReO_3_ undergoes a pressure-induced second-order phase transition at *P*_*c*_ = 0.52 GPa (Axe et al., 1985). Further studies of transport at *T* = 2 K showed that the lowest structural phase boundary terminates at a light hydrostatic pressure of only *P*_*c*_ = 0.25 GPa, observed through a change of Fermi surface cross section (Schirber and Morosin, 1979); however, few reports are available in this difficult *P*-*T* region. Based on early high-temperature data, the pressure-induced phase is likely the tetragonal *P*4/*mbm*, although recent indications of a direct transition to a cubic $Im\bar{3}$ phase have also been reported (Axe et al., 1985; Jørgensen et al., 1986). Neutron diffraction at elevated hydrostatic pressures revealed that the $Im\bar{3}$ phase is stable in the pressure range 0.5 to 13.2 GPa, above which the phase changes to the rhombohedral $R\bar{3}c$ space group (Jørgensen et al., 2004). The soft mode driving the pressure and temperature induced structural transition between the $Pm\bar{3}m$ and $Im\bar{3}$ cubic phases was shown to be three-component $M_{3}^{+}$ phonon mode involving anti-phase rotation of the neighboring ReO_3_ octahedra in an *a*^+^*a*^+^*a*^+^ tilt pattern (Figure 3C). The temperature-dependence of the $M_{3}^{+}$ mode frequency as a function of temperature at ambient pressure is shown in Figure 6B, along with a fit to the mean-field result (Equation 1). This mode is significant in that it is used to understand NTE behavior of open-perovskite systems but is also identified as an order parameter of the phase transition (Chatterji et al., 2009a).

Unlike oxides, fluorides commonly form stable *A*-site-free perovskite structures *B*F_3_ due to the wider array of available *B*^3+^ ion valence configurations among the transition metals. Prominent among these open-perovskite fluorides is ScF_3_, which was discovered in 2010 by Greve et al. (2010) to exhibit a robust NTE effect, which has significant maximal magnitude of the linear CTE α_ℓ_ ≃ −15 ppm/K, persisting over the broad temperature range of 4–1050 K (Figure 5). At room temperature, ScF_3_ crystallizes isostructurally to ReO_3_ with space group symmetry $Pm\bar{3}m$ and has been found to possess related structural instabilitiies corresponding to zone-boundary optical phonons. In ReO_3_, the condensing soft mode responsible for the low-*T* high-*P* structural phase transition is the $M_{3}^{+}$ distortion, while ScF_3_ and other 3*d*-transition metal trifluorides fall into the lower-symmetry rhombohedral $R\bar{3}c$ space group symmetry, attributed to the condensation of the $R_{4}^{+}$ optical phonon.

Although the cubic phase of ScF_3_ is stable at ambient pressure over the entire temperature of the solid phase down to *T* = 0.4K (Romao et al., 2015), X-ray diffraction (Aleksandrov et al., 2009; Greve et al., 2010) and Raman spectroscopy (Aleksandrov et al., 2009) results have revealed that ScF_3_ undergoes several pressure-induced phase transitions. The first is from cubic to rhombohedral (*c*-*r*) after *P*_*c*_ = 0.6 GPa at *T*=300K, with a subsequent rhombohedral to orthorhombic transition occurring above *P*_*c*_ = 3.0 GPa. The *c*-*r* transition has an observed pressure dependence of *dT*_*c*_/*dP* ≃ 525 K/GPa (Aleksandrov et al., 2009, 2011; Greve et al., 2010). Measurement of the lattice dynamics and the soft $R_{4}^{+}$ mode responsible for the rhombohedral transition were performed using inelastic x-ray scattering (IXS), which revealed a 1D manifold of soft optical phonons that circumscribe the entire cubic Brillouin zone-edge. At room temperature, this manifold of modes along *M*-*R* have energy ℏω ≃ 3 meV, softening nearly uniformly to < 1 meV at cryogenic temperatures (see Figure 6B) (Handunkanda et al., 2015). The IXS results combined with structural data permit an estimation that pressures as small as *P*_*c*_ ≃ 0.074 GPa would be sufficient to drive the transition to 0 K. The sensitivity of the phase boundary suggests that the nature of the cubic phase is delicate at low temperature and has been shown to be susceptible to even mild perturbations (Morelock et al., 2013a, 2014, 2015), implying that the ground state of this ionic insulator lie in close proximity to a SQPT.

Phase stability and thermal expansion effects in the open-perovskite trifluoride structure have also been investigated thoroughly through chemical substitution. Chemical substitutions of Sc by Ti (Morelock et al., 2014), Al (Morelock et al., 2015), and Y (Morelock et al., 2013a) have been reported and the effects of this compositional disorder will be discussed in detail in sections 4, 5. Other investigations of changing the stoichiometry have resulted in a related class of hexafluoride compounds, one of which is CaZrF_6_. This material has $Fm\bar{3}m$ space group symmetry and is related to the $Pm\bar{3}m$ structure of ReO_3_ but with a staggered *B*-site ion; that is, alternating CaF_6_ and ZrF_6_ octahedra tiling a simple cubic point-group structure. The resultant (π*ππ*) pattern is likely a key feature when attempting to relate these materials, and is in particular likely to impact the (simple cubic) *M*-*R* BZ edge mode dispersion and dimensional reduction observed in ScF_3_ (Handunkanda et al., 2015). Compared to ScF_3_, this system has isotropic NTE of larger magnitude α_ℓ_ ≃ −18 ppm/K over a temperature range >1050*K* (Figure 5). At *P* = 0 the system also remains cubic at all temperatures above 10 K but a pressure-induced transition to a disordered state occurs near *P*_*c*_ = 0.45 GPa (Hancock et al., 2015), closer in the *P*-*T* diagram than the *c*-*r* transition in ScF_3_ (~0.65 GPa) (Aleksandrov et al., 2003, 2009; Greve et al., 2010). Early computational work suggests the Γ−*X* manifold in the cubic BZ for this compound contribute most strongly to NTE (Gupta et al., 2018), but inelastic scattering measurements of the phonon dynamics are needed to assess the influence of the staggered substitution on the critical SNTE dynamics.

The open-perovskites presented above demonstrate this frameworks' favorable environment for harboring SNTE, but begs the question of why most other purely stoichiometric transition metal trifluorides and perovskites show more conventional thermal expansion. The lattice parameters of each of these SNTE perovskites are plotted in comparison to the prototypical SNTE material ZrW_2_O_8_ in Figure 5, which gives a clear ranking of SNTE perovskites by the magnitude of the NTE effect ((1) CaZrF_6_, (2) ScF_3_, (3) ReO_3_). This ranking is the opposite ordering one gets in terms of pressure required to induce the structural phase transition nearest ambient conditions, a correlation suggestive that proximity to a SQPT and strength of NTE are interrelated. We demonstrate this point for ReO_3_ and ScF_3_ in Figure 6. These plots consider the soft-mode in each system, the $M_{3}^{+}$ phonon in ReO_3_ and the $R_{4}^{+}$ in ScF_3_ and the available data for the energy of these modes as a function of pressure and temperature. First considering the *T*-dependent data at *P* = 0 in Figure 6A, extrapolation of the squared mode energies by Equation (1) provides a quantitative measure of the proximity to a dynamically-driven SPT, yielding *T*_*c*_ ≃ −292K in ReO_3_ (Chatterji et al., 2009a) and *T*_*c*_ ≃ −39K in ScF_3_ (Handunkanda et al., 2015). Furthermore, isothermal measurements of the soft-mode energy vs. hydrostatic pressure are provided in Figure 6B, showing that decreasing temperature and increasing pressure in both systems trend toward a QCP.

Together, the results of these data clearly show in all respects that ScF_3_ is closer to a SQPT than ReO_3_. Although data at this level in unavailable for CaZrF_6_, the amorphization boundary at *P*_*c*_ ≤ 0.45 GPa and *T* = 300K is a lower pressure threshold for the pressure-induced transitions at 300K in both ReO_3_ (~0.55 GPa) and ScF_3_ (~0.65 GPa). It is thus likely that ground-state of this compound is the closest to a structural instability at cryogenic temperature, while also exhibiting the most superlative SNTE effect in this class. Our central hypothesis in the context of the materials described is that the *T* = 0K termination of a structural phase boundary defines a structural quantum critical point (SQCP) where strong geometrical fluctuations associated with octahedral tilts drives NTE. In our view, the significance of the SQCP is a flattening of the energy landscape with respect to transverse fluctuation of the linkage unit: O in ReO_3_, and F in ScF_3_ and CaZrF_6_. It is worth noting that NTE arising from phase fluctuations and the displacements of a low-*T* soft-mode is not unique to the antiferrodistortive (zone-boundary) phonons in perovskites, but has also predicted SNTE in materials with broadly distinct structures and geometrical motifs, e.g., the Hg dimer in Hg_2_I_2_ (Occhialini et al., 2017), the CN molecule in Prussian blue analogs and related compounds (Goodwin et al., 2008; Mittal et al., 2009; Fairbank et al., 2012).

NTE is often understood through the response of the phonon spectrum to the application of hydrostatic pressure, which has been formalized in the quasi-harmonic approximation (QHA) known as the Grüneisen approach (Ashcroft and Mermin, 1976). Each phonon in the Brillouin zone of frequency and wavevector (ω_*i*_, **k**_*i*_) is assigned a mode Grüneisen parameter γ_*i*_, defined as,

(2) $$\begin{array}{r} \gamma_{i}\equiv -\frac{\partial \ln\;\omega_{i}}{\partial \ln\;V}\equiv \frac{1}{\kappa }\frac{\partial \ln\;\omega_{i}}{\partial P} \end{array}$$

where κ is the isothermal compressibility. Performing an average over all wavevectors **k** in the first Brillouin zone, weighted by the mode contribution to the heat capacity *c*_*V, i*_, gives the overall lattice Grüneisen constant γ which is thermodynamically proportional to the volumetric thermal expansion α_*V*_ for isotropic materials. At low-temperatures, the thermodynamic properties are dominated by contributions from the lowest energy excitations. If the low-energy phonon spectrum has large magnitude, negative mode Grüneisen parameters (negative contributions to CTE), then the **k**-averaged CTE will decrease as temperature is lowered. If strong enough to overcome the many high-energy excitations commonly attributed to conventional PTE, the overall expansion may turn negative in sign, strengthening at lower temperature, which is the typical functional form among the SNTE perovskites (Figure 5), before relaxing and limiting to a thermodynamically-required α_*V*_ = 0 as *T* → 0K. From this viewpoint, soft-modes with NTE contributions are natural candidates for inducing overall NTE, since their energy softens with lowering temperature, enhancing the mode occupation and weighted contributions to the thermodynamics at low-*T* in comparison to thermally-stable low energy excitations.

In the SNTE perovskites and other SNTE materials like ZrW_2_O_8_, these lowest energy lattice excitations are commonly attributed to quasi-rigid dynamics of polyhedral subunits (Dove and Fang, 2016; Schlesinger et al., 2008), i.e., the geometrically rigid octahedra as shown in Figure 2 which could correspond to *BX*_6_ octahedra in ScF_3_, ReO_3_ or CaZrF_6_. These rigid unit mode (RUM) analyses model rigidity by freezing out portions of the phonon spectrum, such as high-energy bond-stretch and internal polyhedral bond-bend modes that are commonly attributed to causing PTE. For ScF_3_ and ReO_3_, the antiferrodistortive, zone-edge soft modes have an interpretation as RUMs. Moving beyond the commonly employed QHA and Landau mean-field approaches, we make the hypothesized relationship among soft RUMs, phase fluctuations and the development of SNTE precise within a systematic model in section 4 below.

## 4. Theory of SNTE from RUM fluctuations

The purpose of this section is to present a microscopic description of NTE arising from soft modes in ReO_3_-type lattice structures. Such modes break the symmetry of the lattice and lead to displacive structural phase transitions (Giddy et al., 1993). Typical examples are the $R_{4}^{+}$ mode at the point (1, 1, 1)(π/*a*) of the Brillouin zone of the cubic (c) $Pm\bar{3}m$ phase in MF_3_ (M=Sc, Al, Cr, V, Fe, Ti) metal fluorides which upon condensation gives rise to a rhombohedral (r) $R\bar{3}c$ lattice structure and the $M_{3}^{+}$ mode at (1, 1, 0)(π/*a*) in ReO_3_ which generates a tetragonal (*P*4/*mbm*) phase.

The structural transitions observed in these materials are generally described by Landau theories (Axe et al., 1985; Corrales-Salazar et al., 2017). Typically, they include an order parameter (OP) associated with cooperative tilts of a rigid unit (e.g., the *M*F_6_ octahedron in the metal fluorides) coupled to long-wavelength acoustic phonons that generate volume, deviatoric and shear strains. While such mean field theories provide a fair description of the structural transitions, they fail to describe NTE, e.g., they predict zero thermal expansion in their high-*T* cubic $Pm\bar{3}m$ phase (Corrales-Salazar et al., 2017).

Here, we present a microscopic phenomenology that describes NTE in these open perovskite frameworks. The model includes the usual rigid tilts coupled to long-wavelength strain-generating acoustic modes as well as a cooperative interaction between tilts that drives the structural transition, e.g., dipolar interactions in the metal trifluorides (Chaudhuri et al., 2004; Chen et al., 2004; Allen et al., 2006). Our main result is that any solution of the model must include fluctuations of the OP to generate NTE. We illustrate this within a so-called self-consistent phonon approximation (SCPA) in which single site fluctuations are considered while inter-site fluctuations are neglected. This point has been appreciated before (Volker et al., 2004; Simon and Varma, 2000; He et al., 2010), however, no systematic approach has been constructed so far. In addition, our model allow us to parametrize measured macroscopic quantities in terms of microscopic parameters, which provides guidance for materials design. Our model closely follows those of the well-known antiferrodistortive transitions of SrTiO_3_ and LaAlO_3_ (Feder and Pytte, 1970), with the important distinction that we include hydrostatic pressure and account for compositional disorder. The latter is aimed at describing compounds with tunable NTE through composition such as mixed solid solutions of metal triflurides (Morelock et al., 2013b, 2014, 2015). For concreteness, we will consider a *c*-*r* transition similar to that in Sc_*x*_Ti_1−*x*_F_3_ in which the threefold zone-boundary $R_{4}^{+}$ phonon splits into a low-energy *E*_*g*_ doublet and a high energy *A*_1*g*_ singlet at a transition temperature *T*_*c*_ (Daniel et al., 1990).

Our model analysis is by no means exhaustive. More elaborate descriptions that go beyond the picture of rigid tilts involving, for instance, distortions and translations of such building units are usually needed to describe the observed thermal expansion (Li et al., 2011). Also the observed structural transitions are frequently of first-order, which we do not consider here for the sake of simplicity. Nonetheless, our semi-analytic approach accounts for microscopic aspects of the phonon dynamics and its relation to NTE, and helps identify general trends of the solution. Moreover, it provides the basis to build other frameworks that capture atomistic details such as first-principles-based effective model Hamiltonians (Rabe et al., 2007).

### 4.1. Model hamiltonian

We consider a cubic lattice with *N* sites and choose normal mode coordinates **Q**_*i*_ = (*Q*_*ix*_, *Q*_*iy*_, *Q*_*iz*_) in the unit cell *i*(*i* = 1, 2, …., *N*) associated with the $R_{4}^{+}$ mode, the condensation of which leads to the $R\bar{3}c$ rhombohedral phase. **Q**_*i*_ is proportional to the local displacements generated by the cooperative tilts of the MF_6_ octahedra. In addition, we introduce the strain tensor in Voigt notation ϵ_*iα*_, α = 1, …, 6 in unit cell *i*, which is induced by displacements **u**_*i*_ = (*u*_*ix*_, *u*_*iy*_, *u*_*iz*_) of the centers of mass of the unit cells with respect to the acoustic-branch phonons. In order to determine the optical phonon contribution to the thermal expansion, we must couple the displacements **Q**_*i*_ with strains ϵ_*iα*_, leading to a 3-term Hamiltonian of the form,

(3) $$\begin{array}{r} H=H_{Q}+H_{\epsilon }+H_{Q\epsilon }. \end{array}$$

Here, *H*_*Q*_ accounts for harmonic and anharmonic energy contributions from the soft optical phonon up to quartic order in **Q**_*i*_; *H*_ϵ_ is the strain-induced energy depending on the elasticity through the bulk modulus *C*_*a*_, shear moduli and hydrostatic pressure *P*; and *H*_*Qϵ*_ models the coupling between these displacements and strain degrees of freedom with *g*_*a*_ the coupling constant between the displacements and the volume strain. The explicit form of these terms is given in the Supplementary Material. To solve the statistical mechanical problem posed by the Hamiltonian in Equation (3), we use a variational formulation of a SCPA, in which the temperature and pressure dependence of the phonon energies $\Omega_{\nu },\;\left(\nu =R_{4}^{+},A_{1g},E_{g}\right)$, displacements, strain order parameters and phase fluctuations are determined self-consistently from the minimization of the free energy (Pytte, 1972). We here focus on the main results. The details of the model Hamiltonian and its approximate solution are given in the SM.

### 4.2. Thermal expansion, CTE, and grüneisen parameters

**Figure 7:**
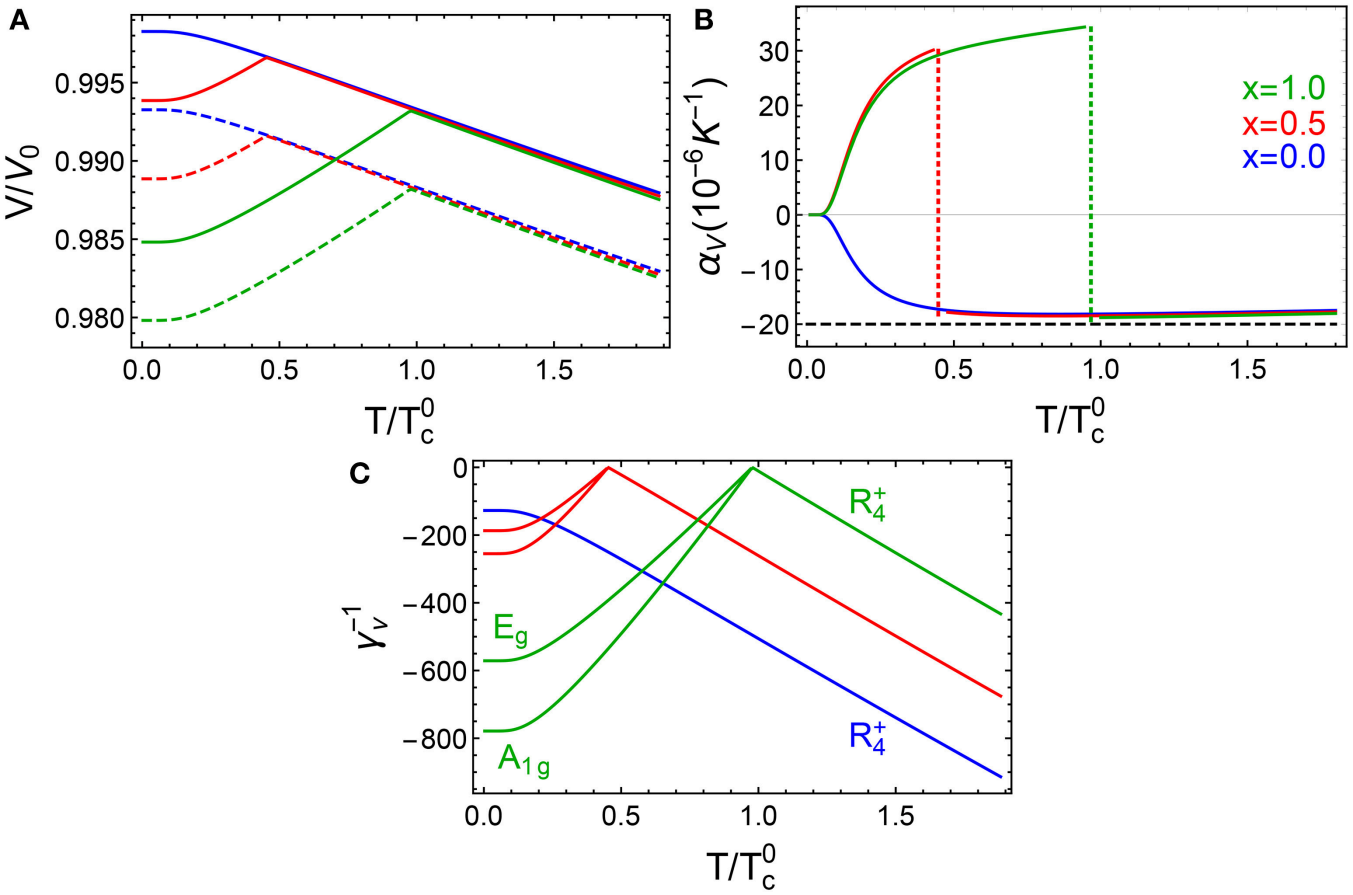
Calculated temperature dependence of **(A)** volume change, **(B)** CTE, and **(C)** Grüneisen parameters for *P*/*P*_0_ = 0 (solid lines) and 9.0 × 10^−5^ (dashed lines) for Sc_1−*x*_Ti_*x*_F_3_. *P*_0_ is the pressure needed to induce the c-r transition in ScF_3_ (*x* = 0) at 0K; $T_{c}^{0}=340$K is the transition temperature of TiF_3_ (*x* = 1) at ambient pressure. Black dashed line in **(B)** is the predicted CTE in the classical limit according to Equation (5). The phonon symmetry labels for *x* = 0.5 in **(C)** are the same as those for *x* = 1 and are not shown for clarity.

We first focus on the volume strain 〈 ϵ_*V*_ 〉 = 〈 ϵ_1_ + ϵ_2_ + ϵ_3_ 〉, which gives the change in volume with temperature and pressure with respect to a reference volume *V*_0_. We use the notation 〈 .…〉 to denote the thermal and compositional disorder average, taken in that order. By minimizing the free energy associated with the Hamiltonian in Equation (3), we find that the volume strain is given as follows,

(4) $$\begin{array}{r} \langle \epsilon_{V}\rangle =\frac{\Delta V}{V_{0}}=-\frac{g_{a}}{C_{a}}\langle |Q{|}^{2}\rangle -\frac{P}{C_{a}}, \end{array}$$

where 〈 |**Q**|^2^〉 is the thermal and compositional average of the squared magnitude of the *M*F_6_ tilt. Equation (4) already illustrates one of the main points of our work: in a mean-field theory and in the absence of pressure, 〈 |**Q**|^2^〉 = 0 above *T*_*c*_; thus fluctuations around the OP must be included to describe NTE. For instance, within the SCPA and for temperatures much greater than the phonon energy, we find that 〈 |**Q**|^2^〉 ∝ *T* in the cubic phase and Equation (4) gives,

(5) $$\begin{array}{r} \frac{\Delta V}{V_{0}}≃\alpha_{V}T-\frac{P}{C_{a}},\;\alpha_{V}=-\frac{3g_{a}k_{B}}{C_{a}v_{R}}, \end{array}$$

where α_*V*_ is the CTE at high temperatures and *v*_*R*_ is the strength of the cooperative interaction. Figures 7A,B show, respectively, our results for the volume change obtained from Equation (4) and its CTE ( α_*V*_ = *d* 〈 ϵ_*V*_〉/*dT*) in the full temperature range. Model parameters were obtained by fitting to experiments (Morelock et al., 2014; Handunkanda et al., 2015) and are given in the SM. Despite its simplicity, our model produces the observed trends (Morelock et al., 2014; Handunkanda et al., 2015): NTE with a nearly linear *T* dependence in the *c*-phase, except near 0K; PTE in the *r*-phase; and a discontinuity in α_*V*_ at the phase transition. Quantitatively, the model is in good agreement in the *c*-phase, but α_*V*_ is about an order of magnitude less than the observed one in the *r*-phase. We attribute this to having neglected the first-order character of the transition and additional phonons along the M-R line of the BZ which are known to contribute to the NTE (van Roekeghem et al., 2016).

We note that Equation (5) gives α_*V*_ in terms of the microscopic model parameters. It shows that mechanically compliant materials with low bulk moduli (*C*_*a*_) and strong strain-phonon couplings (*g*_*a*_) favor thermal expansion. α_*V*_ also increases by weakening the strength of the cooperative interaction *v*_*R*_ at the expense of decreasing the transition temperatures since *T*_*c*_ ∝ *v*_*R*_, as it is shown in the SM. It also shows that the sign of this coupling plays an essential role in the thermal expansion: *g*_*a*_ 〉 0 for NTE while *g*_*a*_ 〈 0 for PTE.

Another physically relevant quantity is the Grüneisen parameter γ_ν_ associated with each lattice mode $\nu =R_{4}^{+},A_{1g},E_{g}$. We find that the temperature and pressure dependence of γ_ν_ is entirely determined by the phonon energy Ω_ν_,

(6) $$\begin{array}{r} \gamma_{\nu }=-\frac{g_{a}}{\Omega_{\nu }^{2}}, \end{array}$$

and thus diverges near the *c*-*r* transition as Ω_ν_ → 0. This is in agreement with previous analytic work (Volker et al., 2004) and ab-initio calculations, where large, negative values for $\gamma_{R_{4}^{+}}$ have been found for ScF_3_ (Li et al., 2011; Liu et al., 2015; van Roekeghem et al., 2016). Figure 7B shows that $\gamma_{\nu }^{-1}\propto -|T-T_{c}|$ at the onset of the phase transition for *x* = 0.5, 1.0 and thus matches the result from Landau theory. For *x* = 0, there is no transition and the deviations from linear behavior are due to zero-point fluctuations.

## 5. The role of disorder in perovskite SNTE materials

Disorder is an inevitable part of any real material system. Here we discuss and develop the role of disorder in on the SNTE effect within the open perovskite structural class.

ReO_3_ has been known as a SNTE material for many years, but there are varying reports of the strength and also extent in temperature over which the effect occurs, which is summarized for recent data by Chatterji (Chatterji and McIntyre, 2006; Chatterji et al., 2009b) and Rodriguez (Rodriguez et al., 2009) in Figure 5 inset. Generally, “open" perovskite oxides are rare due to the requirement of a hexavalent *B*-site and controlled substitutional studies have not been reported to our knowledge. However, the controlled disorder study by Rodriguez (Rodriguez et al., 2009) compared crystals synthesized using different growth techniques and clearly showed that the highest quality crystals grown by chemical vapor transport method exhibited the largest and most thermally persistent SNTE effect. As with the physical properties of many perovskite oxides, controlled post-growth annealing procedure studies may be need to be developed to ensure the optimal NTE effect even in studies of its fundamental causes.

**Figure 8:**
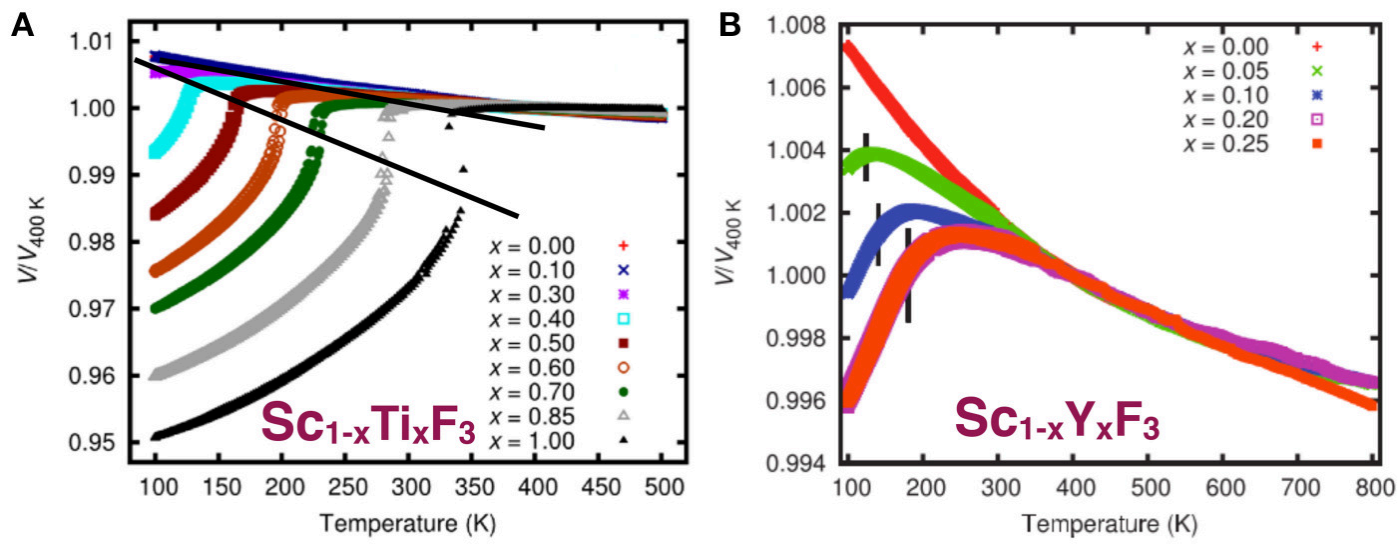
Compositional dependence of the SNTE effect in Sc_1−*x*_(*L* = Ti,Y)_*x*_F_3_ for *L* = Ti (Morelock et al., 2014) and *L* = Y (Morelock et al., 2013b). **(A)** Reprinted with permission from Morelock et al. (2014). Copyright (2018) American Chemical Society. **(B)** Reprinted from Morelock et al. (2013), with permission of AIP publishing.

**Figure 9:**
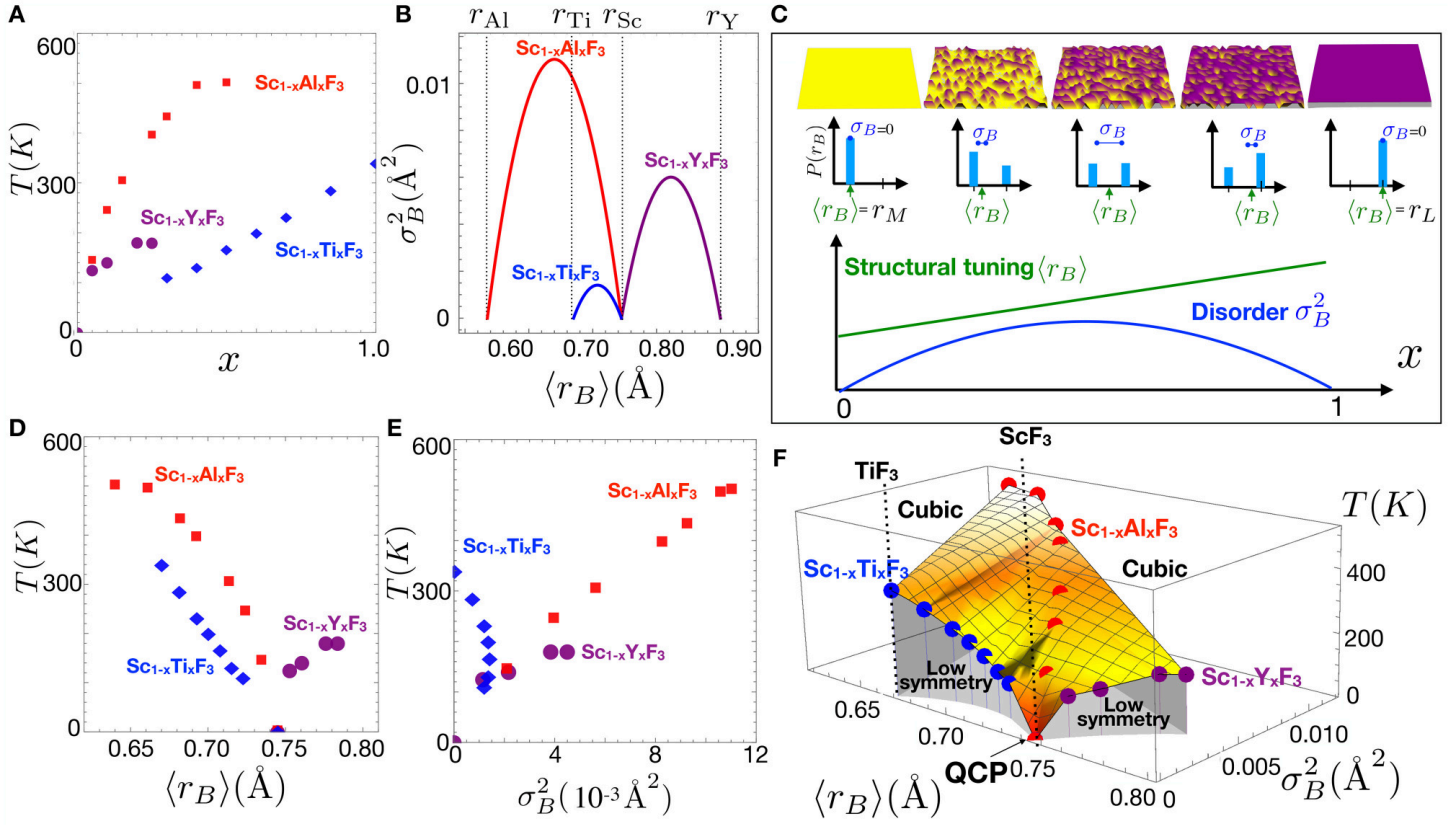
**(A)** Cubic-to-rhombohedral transition temperatures *T*_*c*_(x) in a isovalent substitutional series Sc_1−*x*_*L*_*x*_F_3_. **(B)** The dependence of the mean and variance using Equation (8) and the Shannon ionic radii of the Ti, Y, and Al. (**C**, top row) Illustration of the effect of substitution on the potential energy landscape in a solid solution as the composition *x* is varied. (**C**, middle row) The binary probability distributions *P*(*r*_*B*_) for the five composition levels with the mean and standard deviation σ_*B*_ of the distributions indicated by arrows and dumbbells, respectively. (**C**, bottom) plot of the composition dependence of the first two moments of the distribution according to Equation (8). Plot showing the dependence of the transition temperatures as a function of **(D)** 〈*r*_*B*_〉 and **(E)** $\sigma_{B}^{2}$, respectively, which show no obvious universal trend. **(F)** Combined plot of the transition temperatures vs. the structural tuning parameter 〈*r*_*B*_〉 and the disorder parameter $\sigma_{B}^{2}$.

ScF_3_ is an unusually clean material - single crystals have been synthesized with 0.002 degree mosaic (Handunkanda et al., 2016), free of color centers, with high chemical and isotopic purity with readily available components. The flexibility afforded by the trivalent *B*-site in the trifluorides permits wide chemical tunability and provides new opportunities to observe disorder effects on SNTE. So far, the most thorough and complete studies of the substitutional series Sc_1−*x*_*L*_*x*_F_3_ have been performed with high inorganic synthesis and high quality structural synchrotron and neutron scattering efforts of the Wilkinson group at Georgia Tech. In a series of papers (Morelock et al., 2013b, 2014, 2015), substitutions of *L*=Al,Y,Ti have been reported, particularly the behavior of the cubic-to-rhombohedral phase boundary in this system upon these isovalent substitutions (Figures 8, 9A). Here we develop a combined analysis of these data which permits conclusions regarding the interaction of disorder and the SNTE effect.

Following the spirit of Attfield, who has studied compositional disorder effects on the *A*-site of transition metal oxide phase transitions (Attfield, 1998, 2001, 2002), we borrow the hypothesis that the ionic radius of the substituted ions represents a local energetic influence on the stability of the ordered phase and discuss in our case the probability distribution *P*(*r*_*B*_) of finding a *B*-site ion of radius *r*_*B*_ in the series Sc_1−*x*_*L*_*x*_F_3_. We calculate the first two moments of this distribution and associate the mean ionic radius (1st moment) 〈*r*_*B*_〉 to an energetic effect on the transition and the variance (2nd moment) $\sigma_{B}^{2}$ of the distribution as representative of disorder. For the simple binary distributions shown in Figure 9C, these quantities are simply calculated from the nominal composition *M*_1−*x*_*L*_*x*_F_3_:

(7) $$\begin{array}{rcl} \langle r_{B}\rangle & = & r_{M}\left(1-x\right)+r_{L}x \end{array}$$

(8) $$\begin{array}{rcl} \sigma_{B}^{2} & = & x\left(1-x\right){\left(r_{L}-r_{M}\right)}^{2} \end{array}$$

(9) $$\begin{array}{rc} = & \left(r_{M}-\langle r_{B}\rangle \right)\left(\langle r_{B}\rangle -r_{L}\right). \end{array}$$

These relations are general for any binary mixture, and are applied for *M*=Sc and *L*=Y,Al,Ti in Figure 9B using the Shannon ionic radius for these trivalent ions. Appropriately, $\sigma_{B}^{2}$ is zero for the endpoints of the compositional series and is maximum at the 50-50 composition as expected in all cases. Note that for *L*=Ti, the ion best size matched to Sc, this maximum is small and the effects of disorder are expected to be weaker than for other substitutions, whereas for the much larger Y and much smaller Al ions, disorder increases substantially throughout these series. Further, substitutions of Y have opposite effects on 〈*r*_*B*_〉 than substitutions of Ti and Al, therefore the three substitutional series cover well the transition in terms of both energetics and disorder.

Figures 9D,E show the transition temperatures plotted as a function of 〈*r*_*B*_〉 and $\sigma_{B}^{2}$. There is not a clear common trend in either plot, except that the Y and Al substitution series are linear in $\sigma_{B}^{2}$, implying that quenched disorder is the dominant contribution toward driving the transition, as we have pointed out previously (Handunkanda et al., 2015). For the substitution *L*=Ti, the transition temperature is linear in *x*, suggesting a dominantly energetic effect, as hypothesized based on its similar size and treated theoretically in the weak-disorder limit of the last section. Figure 9F shows a combined plot of all three series *L*=Ti,Al,Y as a function of the structural tuning parameter 〈*r*_*B*_〉 and the disorder parameter $\sigma_{B}^{2}$. This generalized disorder-energy analysis unifies the compositional dependencies of three different series with important implications, showing that disorder is deleterious to SNTE and that ScF_3_ is situated in a very special place which is difficult to reach in the presence of any disorder. These conclusions and the known variation in the SNTE effect of ReO_3_ indicate that disorder generally suppresses the SNTE effect and that careful work optimizing this property with respect to sample history may be necessary in some cases.

ScF_3_ has the most dramatic SNTE effect of all members of these series and also appears at a QCP in the diagram of Figure 9F. Figure 8 reproduces the figure panels for thermal expansion in each series and shows that strong SNTE persists above the transition for light substitutional levels, but weakens in all cases. We point out that no known materials exist in the large 〈*r*_*B*_〉, small $\sigma_{B}^{2}$ limit, but if such a composition could be produced, would be of high interest toward exploring the robustness of SNTE to disorder. Furthermore, routine structural refinement experiments performed at liquid helium temperatures would help immensely toward refining the QCP in these systems where SNTE seems to arise near the *T*=0 termination of a structural phase boundary.

## 6. Summary

We have discussed the broad issue of SNTE with particular focus on perovskite-structured SNTE materials. We have identified the presence of several competing octahedral tilt instabilities occurring near the zero-temperature state of these materials and their associated fluctuations in the high-symmetry phase as key to the SNTE effect in these materials. We have provided a model treatment beyond mean field theory to account for these fluctuations and identified key elements that move toward control of negative thermal expansion and may be invoked for rational design and discovery of future SNTE systems. We also find that quantum mechanical effects are non-negligible and play an important role in SNTE. Finally, we have described existing data in a new analysis which attempts to isolate the influences of energetics and disorder and presented a holistic and generalizable approach leading to the conclusion that disorder disrupts the balance which drives the SNTE effect in ScF_3_, ReO_3_, and other SNTE materials. Our thorough combined analysis of the physical properties and special circumstances in this simple structural class has identified trends and influences that we hope will guide discovery of new SNTE materials.

## Author contributions

CO, SH, and JH wrote sections 1–3 and 5. GG-V developed the modeling in section 4 and the Supplementary Material. All authors contributed to writing and revising the manuscript and figures.

### Conflict of interest statement

The authors declare that the research was conducted in the absence of any commercial or financial relationships that could be construed as a potential conflict of interest.
