# Supplementary material for: Negative Thermal Expansion Near the Precipice of Structural Stability in Open Perovskites
Source: Front Chem. 2018 Nov 20;6:545. doi: 10.3389/fchem.2018.00545 (PMC6255880; doi:10.3389/fchem.2018.00545)
Supplement: Supplementary file 1 [file Data_Sheet_1.pdf]

frontiers

# Supplmentary Material: Negative thermal expansion in open perovskites near the precipice of structural stability

The purpose of this SM is to present the details of our model Hamiltonian and its statistical mechanical solution.

## 1 MODEL HAMILTONIAN

We consider a cubic lattice with $N$ sites and choose normal mode coordinates that describe local displacements $\boldsymbol{Q}_i = (Q_{ix}, Q_{iy}, Q_{iz})$ in the unit cell $i$ $(i = 1, 2, ...., N)$ that are associated with the relevant soft $R_4^+$ phonon mode, the condensation of which leads to a structural transition to a rhombohedral $R\bar{3}c$ phase. In addition, we introduce symmetry adapted strains $\epsilon_{ia} = \epsilon_{i1}+\epsilon_{i2}+\epsilon_{i3}$, $\epsilon_{it} = (2\epsilon_{i3} - \epsilon_{i2} - \epsilon_{i1})/\sqrt{3}$ and $\epsilon_{io} = \epsilon_{i1} - \epsilon_{i2}$, as well as shear strain components $\epsilon_{i4}, \epsilon_{i5}$, and $\epsilon_{i6}$ in the usual Voigt notation: $\epsilon_{i\alpha} = \partial u_{i\alpha}/\partial x_\alpha$ $(\alpha = 1, 2, 3)$, $\epsilon_{i4} = 2\left(\partial u_{iy}/\partial z + \partial u_{iz}/\partial y\right), \epsilon_{i5} = 2\left(\partial u_{ix}/\partial z + \partial u_{iz}/\partial x\right)$, and $\epsilon_{i6} = 2\left(\partial u_{ix}/\partial y + \partial u_{iy}/\partial x\right)$. $\boldsymbol{u}_i = (u_{ix}, u_{iy}, u_{iz})$ is the displacement of the center of mass of the unit cell $i$ from its equilibrium position due to the acoustic phonon mode. Physically, $\epsilon_{ia}$ and $\epsilon_{it/o}$ are, respectively, volume and tetragonal strains. We consider the model Hamiltonian,

$$H = H_Q + H_\epsilon + H_{Q\epsilon}, \tag{S1}$$

where,

$$H_Q = \frac{1}{2}\sum_{i,\lambda}\Pi_{i\nu}^2 + \frac{1}{2}\sum_{i,\lambda}\kappa_i Q_{i\nu}^2 + \frac{\gamma_1}{4}\sum_{i,\lambda\lambda'} Q_{i\lambda}^2 Q_{i\lambda'}^2 + \frac{\gamma_2}{2}\sum_{i,\lambda\neq\lambda'} Q_{i\nu}^2 Q_{i\nu'}^2 - \frac{1}{2}\sum_{ij,\lambda\lambda'} Q_{i\lambda} v_{ij}^{\lambda\lambda'} Q_{j\lambda'}, \tag{S2}$$

$$H_\epsilon = \frac{1}{2}\sum_i |\boldsymbol{\pi}_i|^2 + \frac{1}{2}\sum_i \left[C_a\epsilon_{ia}^2 + C_t\left(\epsilon_{it}^2 + \epsilon_{io}^2\right) + C_r\left(\epsilon_{i4}^2 + \epsilon_{i5}^2 + \epsilon_{i6}^2\right)\right] + P\sum_i \epsilon_{ia}, \tag{S3}$$

and

$$H_{Q\epsilon} = g_a\sum_i \epsilon_{ia}|\boldsymbol{Q}_i|^2 - g_t\sum_i\left[\left(Q_{ix}^2 - Q_{iy}^2\right)\epsilon_{io} + \frac{1}{\sqrt{3}}\left(2Q_{iz}^2 - Q_{iy}^2 - Q_{ix}^2\right)\epsilon_{it}\right] - g_r\sum_i\left(Q_{ix}Q_{iy}\epsilon_{i6} + Q_{ix}Q_{iz}\epsilon_{i5} + Q_{iy}Q_{iz}\epsilon_{i4}\right). \tag{S4}$$

Here, $\boldsymbol{\Pi}_i$ and $\boldsymbol{\pi}_i$ are, respectively, the conjugate momenta of $\boldsymbol{Q}_i$ and $\boldsymbol{u}_i$. $\boldsymbol{v}_{ij}^{\lambda\lambda'}$ $(\lambda, \lambda' = x, y, z)$ is an interaction between the soft mode coordinates with Fourier transform $\boldsymbol{v}_{\boldsymbol{R}+\boldsymbol{q}}^{\lambda\lambda'} = v_{\boldsymbol{R}}\delta_{\lambda\lambda'} + q^2 F_{\lambda\lambda'}(\hat{\boldsymbol{q}})$. This form is typical of cubic lattices with $F_{\lambda\lambda'}(\hat{\boldsymbol{q}})$ dependent on the direction of the unit wave-vector $\hat{\boldsymbol{q}} = \boldsymbol{q}/q$

and independent of the magnitude $q$. Cowley [1980] Within the local SCPA, the equations derived from the stationary property of the free energy are independent of the particular form of $F_{\lambda\lambda'}(\hat{\boldsymbol{q}})$ as long as there is no self-interaction. Pytte [1972] $C_a = (C_{11} + 2C_{12})/3$, is the bulk modulus, and $C_t = (C_{11} - C_{12})/2, C_r = C_{44}$, are deviatoric and shear moduli, respectively. $g_a, g_t$, and $g_r$ are coupling constants between the lattice and the strain degrees of freedom, and $P$ is an applied hydrostatic pressure. $\kappa_i$ is the lattice stiffness at site $i$; $\gamma_1$ and $\gamma_2$ are coefficients of the isotropic and anisotropic cubic anharmonicites, respectively.

To account for quenched compositional disorder in mixed-compounds, we note that at the mean field level the energy barriers between different lattice stuctures depend on the ratio between the harmonic and anharmonic coefficients of the model. Cowley [1980] For simplicity, we thus consider a probability distribution $\mathcal{P}(\kappa_1, \kappa_2, ..., \kappa_N)$ for the $\kappa$'s while assuming that the remaining parameters remain fixed.

To solve the statistical mechanical problem posed by the Hamiltonian (S1), we use a variational formulation of a SCPA in which the energies of the phonon excitations, displacement and strain order parameters are determined from the minimization of the free energy. Pytte [1972]

## 2 STATISTICAL MECHANICAL SOLUTION

We consider the trial probability distribution,

$$\rho^{tr} = \frac{e^{-\beta H^{tr}}}{Z^{tr}}, \tag{S5}$$

where $H^{tr}$ is the Hamiltonian of the local uncoupled problem,

$$H^{tr} = H_Q^{tr} + H_\epsilon^{tr}, \tag{S6}$$

$$H_Q^{tr} = \frac{1}{2}\sum_i |\mathbf{\Pi}_i|^2 + \frac{1}{2}\sum_{i,\alpha\beta} (Q_{i\alpha} - A_{i\alpha}) \mathcal{M}_{\alpha\beta} \left(Q_{i\beta} - A_{i\beta}\right), \tag{S7}$$

$$H_\epsilon^{tr} = \frac{1}{2}\sum_i |\boldsymbol{\pi}_i|^2 + \frac{1}{2}\sum_{i,\alpha\beta} (\epsilon_{i\alpha} - e_{i\alpha}) C_{\alpha\beta} \left(\epsilon_{i\beta} - e_{i\beta}\right), \tag{S8}$$

(S9)

$Z^{tr} = \text{Tr}e^{-\beta H^{tr}}$ is its normalization. $A_{i\alpha} = \langle Q_{i\alpha}\rangle$ and $e_{i\alpha} = \langle e_{i\alpha}\rangle$ are the spontaneous displacement and strain order parameters which will be determined by minimization of the free energy; $\mathcal{M}_{\alpha\beta}$ is the dynamical matrix with eigenfrequencies $\omega_\lambda$ of the non-ineracting problem ($v_{ij}^{\lambda\lambda'} = 0$). Here, $\langle ...\rangle = \text{Tr}\left\{\rho^{tr}....\right\}$ denotes thermal average over the trial probability distribution (S5). We set the long-range ordering associated with the condensation of the $R_4^+$ mode at $R = (1,1,1)(\pi/a)$, by writing $A_{i\alpha} = A_\alpha e^{i\boldsymbol{R}\cdot\boldsymbol{r}_i}$, where $\boldsymbol{r}_i$ is the position vector the lattice site $i$. This corresponds to out-of-phase tilts where $A_{i\lambda}$ changes sign from site to site.

## 2.1 Free energy

The free energy is calculated in the usual way $F = \langle H \rangle + k_B T \left\langle \ln \rho^{tr} \right\rangle$,

$$F = F_Q + F_\epsilon + F_{Q\epsilon}, \tag{S10}$$

where,

$$\begin{aligned} \frac{F_Q}{N} &= \frac{\overline{\kappa}}{2} \langle\, |\boldsymbol{Q}|^2 \,\rangle + \frac{\gamma_1}{4} \langle\, |\boldsymbol{Q}|^4 \,\rangle + \frac{\gamma_2}{2} \left( \left\langle Q_x^2 Q_y^2 \right\rangle + \left\langle Q_x^2 Q_z^2 \right\rangle + \left\langle Q_y^2 Q_z^2 \right\rangle \right) - \frac{1}{2} \sum_{\lambda\lambda'} v_R^{\lambda\lambda'} \langle Q_\lambda \rangle \langle Q_{\lambda'} \rangle \\ &- k_B T \sum_\lambda \left\{ \frac{\beta\omega_\lambda}{2} \coth\left( \frac{\beta\omega_\lambda}{2} \right) - \ln\left[ 2 \sinh\left( \frac{\beta\omega_\lambda}{2} \right) \right] \right\}, \end{aligned} \tag{S11}$$

$$\frac{F_\epsilon}{N} = \frac{1}{2} \left[ C_a e_a^2 + C_t \left( e_t^2 + e_o^2 \right) + C_r \sum_{\nu=4}^{6} e_\nu^2 \right] + P e_a, \tag{S12}$$

and,

$$\begin{aligned} \frac{F_{Q\epsilon}}{N} &= g_a e_a \langle\, |\boldsymbol{Q}|^2 \,\rangle - g_t \left[ \left( \left\langle Q_x^2 \right\rangle - \left\langle Q_y^2 \right\rangle \right) e_o + \frac{1}{\sqrt{3}} \left( 2 \left\langle Q_z^2 \right\rangle - \left\langle Q_x^2 \right\rangle - \left\langle Q_x^2 \right\rangle \right) e_t \right] \\ &- g_r \left[ \langle Q_x Q_y \rangle e_6 + \langle Q_x Q_z \rangle e_5 + \langle Q_y Q_z \rangle e_4 \right], \end{aligned} \tag{S13}$$

with

$$\langle Q_\lambda Q_{\lambda'} \rangle = A_\lambda A_{\lambda'} + \psi_{\lambda\lambda'}, \tag{S14a}$$

$$\left\langle Q_\lambda^2 Q_{\lambda'}^2 \right\rangle = A_\lambda^2 A_{\lambda'}^2 + A_\lambda^2 \psi_{\lambda'\lambda'} + 4 A_\lambda A_{\lambda'} \psi_{\lambda\lambda'} + A_{\lambda'}^2 \psi_{\lambda\lambda} + \psi_{\lambda\lambda} \psi_{\lambda'\lambda'} + 2 \psi_{\lambda\lambda'}^2. \tag{S14b}$$

$\psi_{\lambda\lambda'}$ are the local OP fluctuations given as follows,

$$\psi_{\lambda\lambda'} = \sum_\nu b_{\lambda\nu}^\dagger b_{\nu\lambda'} \left( \frac{1}{2\omega_\nu} \coth\left( \frac{\beta\omega_\nu}{2} \right) \right), \tag{S15}$$

where $b_{\nu\lambda'}$ is an unitary transformation that diagonalizes $\mathcal{M}_{\alpha\beta}$. $\overline{\kappa}$ the lattice stiffness averaged over compositional disorder. In writing Eq. (S10), we have ignored all terms that do not depend on $A_\lambda$, $e_\alpha$, and $\omega_\lambda$ as they do not have an effect on the miminization procedure.

Minimization of the free energy (S10) with respect to the strains $e_\alpha$ gives the following result,

$$e_a = -\frac{g_a}{C_a} \langle\, |\boldsymbol{Q}|^2 \,\rangle - \frac{P}{C_a}, \tag{S16a}$$

$$e_t = \frac{g_t}{\sqrt{3} C_t} \left( 2 \left\langle Q_z^2 \right\rangle - \left\langle Q_x^2 \right\rangle - \left\langle Q_y^2 \right\rangle \right), \tag{S16b}$$

$$e_o = \frac{g_t}{C_t} \left( \left\langle Q_x^2 \right\rangle - \left\langle Q_y^2 \right\rangle \right), \tag{S16c}$$

$$e_4 = \frac{g_r}{C_r} \langle Q_y Q_z \rangle, \quad e_5 = \frac{g_r}{C_r} \langle Q_x Q_z \rangle, \quad e_6 = \frac{g_r}{C_r} \langle Q_x Q_y \rangle. \tag{S16d}$$

By substituting the strains of Eq. (S16) into the Eq. (S10), we obtain a free energy which depends on the displacements $\boldsymbol{Q}$ only, where,

$$
\begin{aligned}
\frac{\tilde{F}}{N} = & \frac{\tilde{\kappa}}{2} \langle\, |\boldsymbol{Q}|^2 \,\rangle + \frac{\gamma_1}{4} \langle\, |\boldsymbol{Q}|^4 \,\rangle + \frac{\gamma_2}{2} \left( \langle Q_x^2 Q_y^2 \rangle + \langle Q_x^2 Q_z^2 \rangle + \langle Q_y^2 Q_z^2 \rangle \right) - \frac{1}{2} \sum_{\lambda\lambda'} v_R^{\lambda\lambda'} \langle Q_\lambda Q_{\lambda'} \rangle \,, \\
& - k_B T \sum_\lambda \left\{ \frac{\beta\omega_\lambda}{2} \coth\left(\frac{\beta\omega_\lambda}{2}\right) - \ln\left[ 2\sinh\left(\frac{\beta\omega_\lambda}{2}\right)\right] \right\}, \\
& - \left( \frac{3}{2}\frac{g_a^2}{C_a} + \frac{2}{3}\frac{g_t^2}{C_t} \right) \left( \langle Q_x^2 \rangle + \langle Q_y^2 \rangle + \langle Q_z^2 \rangle \right)^2 \\
& + \frac{2g_t^2}{C_t} \left( \langle Q_x^2 \rangle \langle Q_y^2 \rangle + \langle Q_x^2 \rangle \langle Q_z^2 \rangle + \langle Q_y^2 \rangle \langle Q_z^2 \rangle \right) \\
& - \frac{1}{2}\frac{g_r^2}{C_r} \left( \langle Q_x Q_y \rangle^2 + \langle Q_x Q_z \rangle^2 + \langle Q_y Q_z \rangle^2 \right) - \frac{P^2}{2C_a}, \qquad \text{(S17)}
\end{aligned}
$$

where $\tilde{\kappa} \equiv \overline{\kappa} - 2g_a P/C_a$. The free energies (S10) & (S17) are the starting point for our calculation of the thermodynamic quantities of interest.

## 2.2 Soft Mode Frequencies

The soft mode frequencies are computed from the free energy (S10) with the $e_\alpha$ constant and then must be evaluated at the equilibrium points given in Eq (S16). This is because the frequency of the acoustic modes associated with uniform strains vanishes in the long-wavelength limit. Slonczewski and Thomas [1970]

The dynamical matrix $\mathcal{D}_{\lambda\lambda'}$ of the interacting problem is calculated from the free energy (S10),

$$\begin{aligned}
\mathcal{D}_{xx} = \frac{\partial^2 \langle H\rangle}{\partial A_x \partial A_x} &= \kappa + (2\gamma_1 - \gamma_2)\left\langle Q_x^2\right\rangle + (\gamma_1+\gamma_2)\left\langle\, |\boldsymbol{Q}|^2\,\right\rangle \\
&\quad + 2g_a e_a - 2g_t\left(e_o - \frac{e_t}{\sqrt{3}}\right) - v_R, \\
\mathcal{D}_{yy} = \frac{\partial^2 \langle H\rangle}{\partial A_y \partial A_y} &= \kappa + (2\gamma_1 - \gamma_2)\left\langle Q_y^2\right\rangle + (\gamma_1+\gamma_2)\left\langle\, |\boldsymbol{Q}|^2\,\right\rangle \\
&\quad + 2g_a e_a + 2g_t\left(e_o + \frac{e_t}{\sqrt{3}}\right) - v_R, \\
\mathcal{D}_{zy} = \frac{\partial^2 \langle H\rangle}{\partial A_z \partial A_z} &= \kappa + (2\gamma_1 - \gamma_2)\left\langle Q_z^2\right\rangle + (\gamma_1+\gamma_2)\left\langle\, |\boldsymbol{Q}|^2\,\right\rangle \\
&\quad + 2g_a e_a - 4g_t\frac{e_t}{\sqrt{3}} - v_R, \\
\mathcal{D}_{xy} = \frac{\partial^2 \langle H\rangle}{\partial A_x \partial A_y} &= 2\left(\gamma_1+\gamma_2\right)\langle Q_x Q_y\rangle - g_r e_6, \\
\mathcal{D}_{xz} = \frac{\partial^2 \langle H\rangle}{\partial A_x \partial A_z} &= 2\left(\gamma_1+\gamma_2\right)\langle Q_x Q_z\rangle - g_r e_5, \\
\mathcal{D}_{yz} = \frac{\partial^2 \langle H\rangle}{\partial A_y \partial A_z} &= 2\left(\gamma_1+\gamma_2\right)\langle Q_y Q_z\rangle - g_r e_4,
\end{aligned}$$

where $\langle Q_\lambda Q_{\lambda'}\rangle$ is given by Eq. (S14). To proceed further, we now consider the cubic and rhombohedral phases separately.

## 2.3 Cubic phase

In the cubic phase, $A_x = A_y = A_z = 0, \left\langle Q_x^2\right\rangle = \left\langle Q_y^2\right\rangle = \left\langle Q_z^2\right\rangle = \psi_0, \langle Q_x Q_y\rangle = \langle Q_x Q_z\rangle = \langle Q_y Q_z\rangle = 0, e_a \neq 0, e_t = e_o = e_4 = e_5 = e_6 = 0, b_{\lambda\lambda'} = \delta_{\lambda\lambda'}$. Thus, the dynamical matrix is given as follows,

$$\begin{aligned}
&\mathcal{D}_{xx} = \mathcal{D}_{yy} = \mathcal{D}_{zz} = \overline{\kappa} + (5\gamma_1 + 2\gamma_2)\,\psi_0 + 2g_a e_a - v_R, \\
&\mathcal{D}_{xy} = \mathcal{D}_{xz} = \mathcal{D}_{yz} = 0.
\end{aligned}$$

The diagonalization of $\mathcal{D}_{\lambda\lambda'}$ gives a triply degenerate zone-boundary soft mode frequency,

$$\Omega^2_{R_4^+} = \omega^2_{R_4^+} + (5\gamma_1 + 2\gamma_2)\,\psi_0 + 2g_a e_a. \tag{S18}$$

Here, $\omega_{R_4^+} \equiv \sqrt{\overline{\kappa} - v_R}$ is the frequency of a purely harmonic model. $\psi_0$ are the local OP fluctuations in the cubic phase,

$$\psi_0 = \frac{1}{2\omega_R} \coth\left(\frac{\beta\omega_R}{2}\right), \tag{S19}$$

with $\omega_R = \sqrt{\Omega^2_{R_4^+} + v_R}$. The change in volume is given by the volumetric strain,

$$\frac{\Delta V}{V_0} = e_a = -\frac{3g_a}{C_a}\psi_0 - \frac{P}{C_a}, \tag{S20}$$

where $V_0$ is a reference volume.

We now calculate the Grüneisen parameter associated with $R_4^+$. From Eq. (S18), we find that the temperature and pressure dependence of $\gamma_{R_4^+}$ is entirely determined by the phonon energy, Volker et al. [2004a]

$$\gamma_{R_4^+} = -\frac{\partial \ln \Omega_{R_4^+}}{\partial e_a} = -\frac{g_a}{\Omega^2_{R_4^+}}. \tag{S21}$$

Equations (S18)-(S20), determine self-consistently the temperature and pressure dependence of $\Omega_{R_4^+}$, and $e_a$.

## 2.4 Rhombohedral phase

In the r-phase, $A_x = A_y = A_z = A/\sqrt{3}, \psi_1 \equiv \psi_{xx} = \psi_{yy} = \psi_{zz}, \psi_4 \equiv \psi_{xy} = \psi_{xz} = \psi_{yz}, e_a \neq 0, e_o = e_t = 0, e_r \neq 0$ and,

$$b_{\lambda\lambda'} = \begin{pmatrix} \frac{1}{\sqrt{6}} & \frac{1}{\sqrt{2}} & \frac{1}{\sqrt{3}} \\ \frac{1}{\sqrt{6}} & -\frac{1}{\sqrt{2}} & \frac{1}{\sqrt{3}} \\ -\frac{2}{\sqrt{6}} & 0 & \frac{1}{\sqrt{3}} \end{pmatrix}. \tag{S22}$$

Thus, the dynamical matrix is given as follows,

$$\mathcal{D}_{xx} = \mathcal{D}_{yy} = \mathcal{D}_{zz} = \overline{\kappa} + (2\gamma_1 - \gamma_2)\left(\frac{A^2}{3} + \psi_1\right) + (\gamma_1 + \gamma_2)(A + 3\psi_1) + 2g_a e_a - v_R,$$

$$\mathcal{D}_{xy} = \mathcal{D}_{xz} = \mathcal{D}_{yz} = 2(\gamma_1 + \gamma_2)\left(\frac{A^2}{3} + \psi_4\right) - g_r e_r.$$

The diagonalization of $\mathcal{D}_{\lambda\lambda}$ together with the minimization of the free energy (S17) with respect to $A$ gives the following result,

$$\Omega_{E_g}^2 = \omega_{R_4^+}^2 + (5\gamma_1 + 2\gamma_2)\left(\frac{A^2}{3} + \psi_1\right) + 2g_a e_a - 2(\gamma_1 + \gamma_2)\left(\frac{A^2}{3} + \psi_4\right) + g_r e_r, \quad \text{(S23a)}$$

$$\Omega_{A_{1g}}^2 = \omega_{R_4^+}^2 + (5\gamma_1 + 2\gamma_2)\left(\frac{A^2}{3} + \psi_1\right) + 2g_a e_a + 4(\gamma_1 + \gamma_2)\left(\frac{A^2}{3} + \psi_4\right) - 2g_r e_r, \quad \text{(S23b)}$$

$$\Omega_{A_{1g}}^2 = 2(3\gamma_1 + 2\gamma_2)\frac{A^2}{3}, \quad \text{(S23c)}$$

$e_a$ and $e_r$ are volume and shear strains, respectively

$$e_a = -\frac{3g_a}{C_a}\left(\frac{A^2}{3} + \psi_1\right) - \frac{P}{C_a}, \quad \text{(S24a)}$$

$$e_r = \frac{g_r}{C_r}\left(\frac{A^2}{3} + \psi_4\right). \quad \text{(S24b)}$$

$\psi_1$ and $\psi_4$ are fluctuations of the OP in the r-phase,

$$\psi_1 = \frac{1}{3}\left(\frac{1}{2\omega_{A_{1g}}}\coth\left(\frac{\beta\omega_{A_{1g}}}{2}\right) + \frac{1}{\omega_{E_g}}\coth\left(\frac{\beta\omega_{E_g}}{2}\right)\right), \quad \text{(S25a)}$$

$$\psi_4 = \frac{1}{3}\left(\frac{1}{2\omega_{A_{1g}}}\coth\left(\frac{\beta\omega_{A_{1g}}}{2}\right) - \frac{1}{2\omega_{E_g}}\coth\left(\frac{\beta\omega_{E_g}}{2}\right)\right), \quad \text{(S25b)}$$

where $\omega_{E_g,A_{1g}} = \sqrt{\Omega_{E_g,A_{1g}}^2 + v_R}$.

As mentioned above, the temperature and pressure dependence of the the Grüneisen parameters of the $E_g$ and $A_{1g}$ phonons are again entirely determined by their corresponding energies,

$$\gamma_{E_g,A_{1g}} = -\frac{\partial \ln \Omega_{E_g,A_{1g}}}{\partial e_a} = -\frac{g_a}{\Omega_{E_g,A_{1g}}^2}. \quad \text{(S26)}$$

Equations (S23)-(S25), determine self-consistently the temperature and pressure dependence of $\Omega_{E_g/A_{1g}}$, $A$, $e_r$ and $e_a$.

### 2.4.1 Classical Limit

It is useful to consider the classical limit of the above results, as it allow us to derive analytical expressions for several relevant macroscopic quantities in terms of microscopic parameters. We consider the high-$T$ cubic phase.

In the classical limit ($\beta\omega \ll 1$) and near the structural transition, $\psi_0 \simeq k_B T/v_R$, thus,

$$\frac{\Delta V}{V_0} \simeq \alpha_V T - \frac{P}{C_a}, \quad \alpha_V = -\frac{3g_a k_B}{C_a v_R} \quad \text{(S27)}$$

where $\alpha_V$ is the coefficient of thermal expansion (CTE).

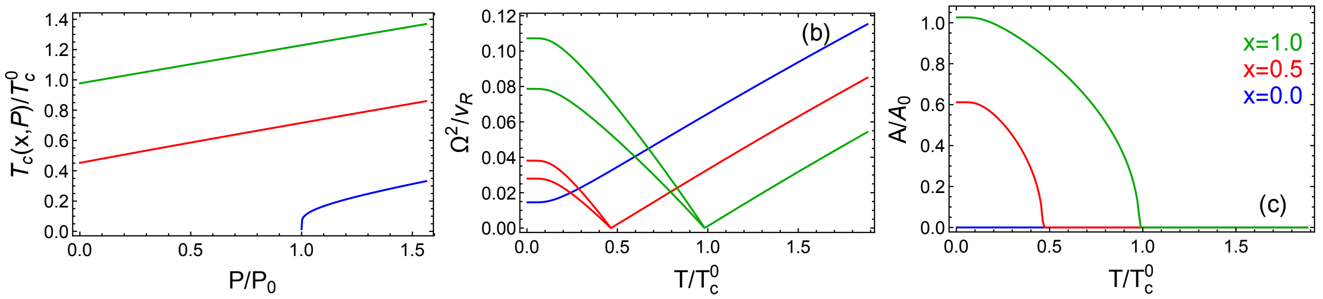


Figure S1: Calculated (a) phase diagram (b) soft mode frequencies and (c) order parameter for $Sc_{1-x}Ti_xF_3$.

We find that he $T$ and $P$ dependence of $\Omega_{R_4^+}$ matches that of Landau theory, as expected, Pytte [1972]

$$\Omega_{R_4^+}^2 = -\omega_{R_4^+}^2 t, \tag{S28}$$

where $t = \frac{T - T_c(x,P)}{T_c(x,P)}$ is a reduced temperature and $T_c(x, P)$ is a pressure dependent transition temperature given as follows,

$$T_c(x, P) = T_c(x, 0) + \frac{2g_a/C_a}{\gamma k_B/v_R} P, \tag{S29}$$

where $T_c(x, 0)$ is the transiton temperature at ambient pressure,

$$T_c(x, 0) = -\frac{\omega_{R_4^+}^2}{\gamma k_B/v_R} \tag{S30}$$

with $\gamma \equiv 5\gamma_1 + 2\gamma_2 - 6g_a^2/C_a$. Note that when there is a c-r transition, the high symmetry phase is unstable in the purely harmonic approximations and thus $\omega_{R_4^+}^2 < 0$. The $x$ dependence in $T_c\,(x, P)$ is through $\omega_{R_4^+}$, which in turn depends $\overline{\kappa}$. Note that for $g_a > 0$, hydrostatic pressure destabilizes the c-phase. From Eq. (S29), we find a proportionality relation between the slope of the $T - P$ phase diagram and the CTE,

$$\frac{dT_c}{dP} = \frac{2g_a/C_a}{\gamma k_B/v_R} \propto -\alpha_V. \tag{S31}$$

We conclude the presentation of our model here.

## 3 MODEL PARAMETERS

The model parameters are given in Table S1 and were obtained from fits to experiments in $Sc_xTi_{1-x}F_3$. Handunkanda et al. [2015]; Morelock et al. [2014] We have assumed independent bimodal distributions for the stiffnesses $\mathcal{P}(\kappa_1, \kappa_2, ..., \kappa_N) = \prod_{i=1}^{N} \mathcal{P}(\kappa_i)$, with $\mathcal{P}(\kappa_i) = x\delta(\kappa_i - \kappa_{\mathrm{Ti}}) + (1-x)\,\delta(\kappa_i - \kappa_{\mathrm{Sc}})$ and $\kappa_{\mathrm{Ti/Sc}}$ are the lattice stiffnesses of the pure compounds. With this choice, $\overline{\kappa} = x\kappa_{\mathrm{Ti}} + (1-x)\,\kappa_{\mathrm{Sc}}$. The resulting $T - P$ phase diagram, phonon frequencies and order parameter for several compositions are shown in Fig. S1.

**Table S1.** Model parameters

| | |
|---|---|
| $\kappa_{\mathrm{Ti}}$ [meV$^2$] | 161 |
| $\kappa_{\mathrm{Sc}}$ [meV$^2$] | 173 |
| $v_R$ [ meV$^2$] | 173 |
| $\gamma_1$ [ meV$^3$] | 21.8 |
| $\gamma_2$ [ meV$^3$] | $-19.4$ |
| $g_a$ [ meV$^2$] | 0.023 |
| $g_r$ [ meV$^2$] | 0.019 |
| $C_a$ [ meV] | 1.0 |
| $C_r$ [ meV] | 0.22 |

## REFERENCES

Cowley, R. A. (1980). Structural phase transitions I. Landau theory. *Adv. Phys.* 29, 1

Handunkanda, S. U., Curry, E. B., Voronov, V., Said, A. H., Guzmán-Verri, G. G., Brierley, R. T., et al. (2015). Large isotropic negative thermal expansion above a structural quantum phase transition. *Phys. Rev. B* 92, 134101. doi:10.1103/PhysRevB.92.134101

Morelock, C. R., Gallington, L. C., and Wilkinson, A. P. (2014). Evolution of negative thermal expansion and phase transitions in $Sc_{1-x}Ti_xF_3$. *Chem. Mater.* 26, 1936. doi:10.1021/cm5002048

Pytte, E. (1972). Theory of perovskite ferroelectrics. *Phys. Rev. B* 5, 3758–3769. doi:10.1103/PhysRevB.5.3758

Slonczewski, J. C. and Thomas, H. (1970). Interaction of elastic strain with the structural transition of strontium titanate. *Phys. Rev. B* 1, 3599. doi:10.1103/PhysRevB.1.3599

Volker, H., Welche, P. R. L., and Dove, M. T. (2004a). Geometrical origin and theory of negative thermal expansion in framework structures. *Journal of the American Ceramic Society* 82, 1793. doi:10.1111/j.1151-2916.1999.tb02001.x
